# Astrocyte-derived lactate aggravates brain injury of ischemic stroke in mice by promoting the formation of protein lactylation

**DOI:** 10.7150/thno.96375

**Published:** 2024-07-08

**Authors:** Xiao-Yi Xiong, Xin-Ru Pan, Xia-Xia Luo, Yu-Fei Wang, Xin-Xiao Zhang, Su-Hao Yang, Zhan-Qiong Zhong, Chang Liu, Qiong Chen, Peng-Fei Wang, Xiao-Wei Chen, Shu-Guang Yu, Qing-Wu Yang

**Affiliations:** 1 Acupuncture and Tuina School, Chengdu University of Traditional Chinese Medicine; 1166 Liutai Road, 611137, Chengdu, China.; 2 Sichuan Provincial Key Laboratory for Acupuncture & Chronobiology, Chengdu, China.; 3 Key Laboratory of Acupuncture for Senile Disease (Chengdu University of TCM), Ministry of Education, Chengdu, China.; 4 School of Basic Medical Sciences, Chengdu University of Traditional Chinese Medicine, 1166 Liutai Road, 611137, Chengdu, China.; 5 Department of Neurology, Xinqiao Hospital, the Army Medical University (Third Military Medical University), Chongqing, China.; 6 Department of Neurology, Weihai Municipal Hospital, Weihai, China.; 7 Brain Research Center and State Key Laboratory of Trauma, Burns, and Combined Injury, the Army Medical University (Third Military Medical University), Chongqing, China.

**Keywords:** brain injury, cerebral ischemia, glial activation, lactate, lactylation

## Abstract

**Aim:** Although lactate supplementation at the reperfusion stage of ischemic stroke has been shown to offer neuroprotection, whether the role of accumulated lactate at the ischemia phase is neuroprotection or not remains largely unknown. Thus, in this study, we aimed to investigate the roles and mechanisms of accumulated brain lactate at the ischemia stage in regulating brain injury of ischemic stroke.

**Methods and Results:** Pharmacological inhibition of lactate production by either inhibiting LDHA or glycolysis markedly attenuated the mouse brain injury of ischemic stroke. In contrast, additional lactate supplement further aggravates brain injury, which may be closely related to the induction of neuronal death and A1 astrocytes. The contributing roles of increased lactate at the ischemic stage may be related to the promotive formation of protein lysine lactylation (Kla), while the post-treatment of lactate at the reperfusion stage did not influence the brain protein Kla levels with neuroprotection. Increased protein Kla levels were found mainly in neurons by the HPLC-MS/MS analysis and immunofluorescent staining. Then, pharmacological inhibition of lactate production or blocking the lactate shuttle to neurons showed markedly decreased protein Kla levels in the ischemic brains. Additionally, *Ldha* specific knockout in astrocytes (*Aldh1l1*^CreERT2^; *Ldha*^fl/fl^ mice, cKO) mice with MCAO were constructed and the results showed that the protein Kla level was decreased accompanied by a decrease in the volume of cerebral infarction in cKO mice compared to the control groups. Furthermore, blocking the protein Kla formation by inhibiting the writer p300 with its antagonist A-485 significantly alleviates neuronal death and glial activation of cerebral ischemia with a reduction in the protein Kla level, resulting in extending reperfusion window and improving functional recovery for ischemic stroke.

**Conclusion:** Collectively, increased brain lactate derived from astrocytes aggravates ischemic brain injury by promoting the protein Kla formation, suggesting that inhibiting lactate production or the formation of protein Kla at the ischemia stage presents new therapeutic targets for the treatment of ischemic stroke.

## Introduction

Ischemic brain injury initiated by metabolic disorder is one of the major differences of ischemic stroke from other brain diseases. Physiologically, lactate act as one of the major fuels for neuronal mitochondrial oxidative phosphorylation (OXPHO) to produce ATP. In contrast, glial cells, such as astrocytes, can produce lactate to supply neurons via glycolysis 1. Because the glycolysis pathway does not rely on oxygen, and astrocytes, in which glycogen is mainly localized within the brain 2, can store a certain amount of glycogen; therefore, astrocytic glycolysis, theoretically, becomes a major energy-producing entity during cerebral ischemia that may support glial cell activation after cerebral ischemia 3. However, the accumulated lactate cannot fuel the neurons during ischemia because OXPHO processes are blocked under hypoxia, as evidenced by the substantially decreased brain ATP levels in contrast to the increased lactate levels 4. Interestingly, it has been shown that lactate can impair adult hippocampal neurogenesis although without defined mechanisms 5, further indicating that lactate is emerging as an important signaling molecule and is not merely a by-product of glycolysis and energy source 6. However, although researches have shown that lactate supplementation at the reperfusion stage offers neuroprotection for ischemic stroke, which most likely by acting as an energy substrate 7. However, whether the role of accumulated lactate at the ischemia phase is neuroprotection or not remains largely unknown because lactate cannot be further metabolized to produce energy under the conditions of absence of oxygen.

Evidence has shown that metabolites could act as reaction substrates for protein post-translational modifications (PTMs), which have been observed as some of the most efficient biological mechanisms for regulating cellular pathophysiology 8, 9. For example, acetyl-CoA serves as the primary source for Lys acetylation 10 and succinate for succinylation 11. Recent studies have found that lactate is also an important substrate for the newly identified PTM, lysine lactylation (Kla) 12, 13. These studies have described the key roles of lactate-induced Kla, which serves as an epigenetic modification that directly stimulates gene transcription from chromatin in macrophages 12 and involves the glycolytic pathways 13 and pathogenesis of lung fibrosis 14. These recent results strongly suggest that lactate may also via driving the formation of protein Kla to participate in the regulation of brain injury at the ischemia stage of ischemic stroke under conditions of not being used as material fuel. Therefore, whether the protein Kla is also involved in regulating the brain damage during ischemia stage remains to be investigated although some studies have investigated their roles in regulating the brain injury at the reperfusion stage (the cerebral ischemia/reperfusion injury) 15-17.

In this study, we aimed to investigate the roles and mechanisms of accumulated lactate at the ischemia stage in regulating brain injury of ischemic stroke. We found that increased brain lactate levels derived from astrocytes markedly aggravate the brain injury of ischemic stroke by promoting the formation of protein Kla. Pharmacological inhibition of the lactate production, blockade of the lactate shuttle to neurons, and the formation of protein Kla significantly alleviated the brain injury of ischemic stroke in mice with a reduction of protein Kla levels. Our study reveals, for the first time, the roles and mechanisms of astrocytic lactate-derived protein Kla contribute to ischemic brain injury and provides a novel therapeutic target to improve functional recovery after ischemic stroke.

## Results

### Reducing the lactate production by inhibiting LDHA or glycolytic pathway alleviates brain injury after ischemic stroke

Ischemic brain injury is initiated mainly by metabolic disorders because the sudden blockage of blood vessels disrupts normal glucose metabolism in brain tissue. Increasing evidence shows that an abnormal accumulation of lactate was found both in animals 4, 18, 19 and humans with ischemic stroke 20-22. Although lactate supplementation immediately after reperfusion has been shown to offer neuroprotection for ischemic stroke (Figure S1A) 7, which may be related to their usage as an energy substrate with normal oxygen tension 7. However, the increased brain lactate levels were mainly found at the ischemia stages of ischemic stroke (Figure S1B). Therefore, in this study, we doubted whether the increased brain lactate levels would also offer neuroprotection for ischemic stroke as the lactate acting as neuronal fuel needs normal oxygen tension.

**Figure 1:**
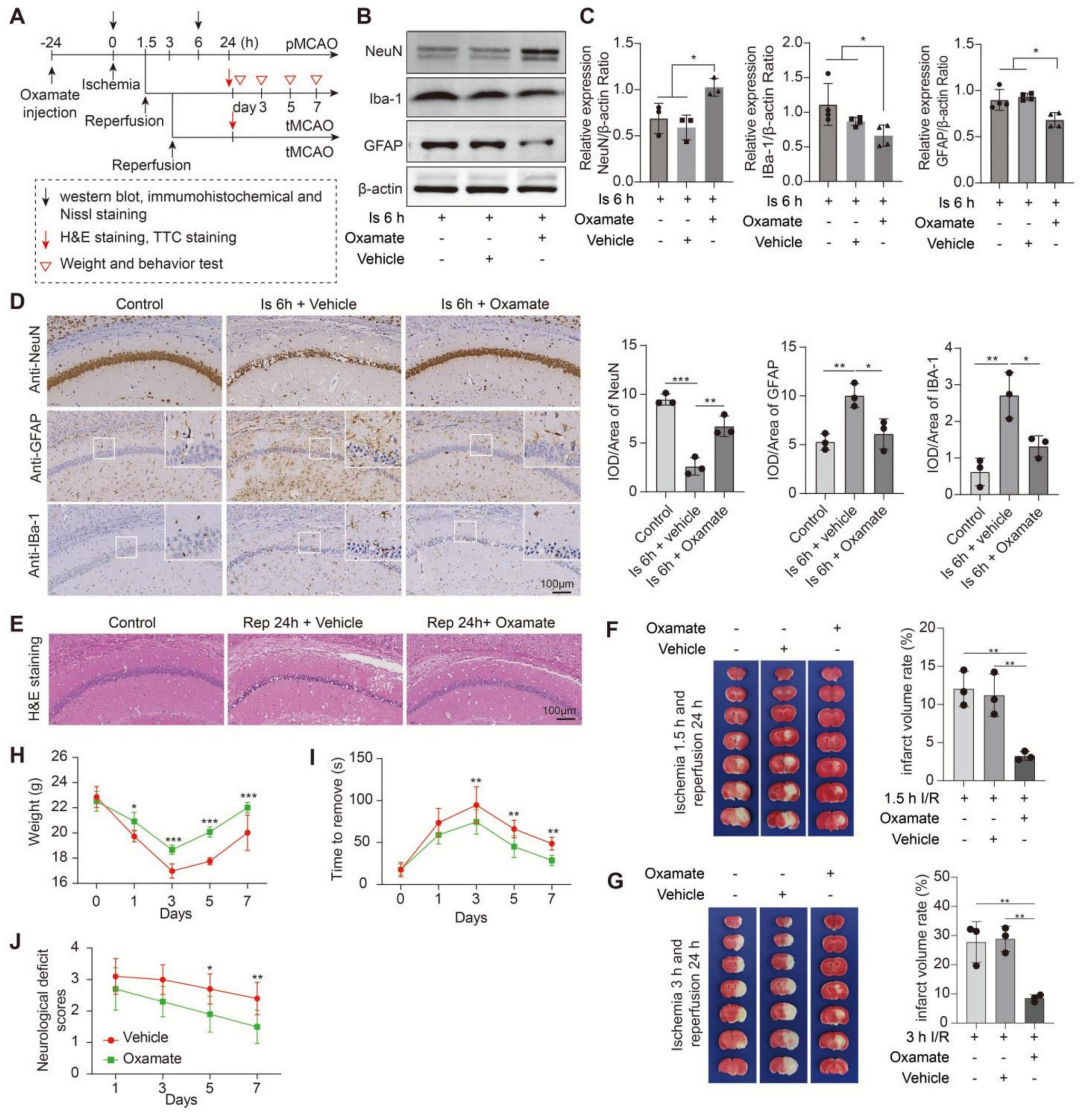
** Inhibition of brain lactate production attenuates brain injury after ischemic stroke. A,** Experimental design and timeline of ischemic stroke, Oxamate treatment, and analysis in mice. pMCAO and tMCAO indicate permanent and transient middle cerebral artery occlusion, respectively. **B-C,** The ischemic brain levels of neuroglial markers of NeuN, GFAP, and IBa-1 were measured using western blot analysis of tissues at 6 h after cerebral ischemia after Oxamate preconditioning (n = 3-4). **D,** Representative images and statistical analysis of immunohistochemical staining show the expression levels of neuroglial markers of NeuN, GFAP, and IBa-1 in the ischemic hippocampus CA1 regions at 6 h after cerebral ischemia after Oxamate preconditioning (n = 3). **E,** Representative images of hematoxylin and eosin (H&E) staining show damage of the ischemic hippocampus CA1 regions at 24 h after reperfusion and 1.5 h of ischemia with Oxamate preconditioning (n = 3). **F-G,** Representative images of 2,3,5-triphenyltetrazolium chloride (TTC) staining of brain sections at 24 h after reperfusion and 1.5 h (F) and 3 h (G) after ischemia. The infarction area is white (n = 3). **H-J,** Mice with Oxamate preconditioning treatment and ischemic stroke show better functional recovery in tests of body weight (H), sensory neglect (I), and neurological deficit scores (J). n = 12. Two-way ANOVA with repeated measures reported a significant effect of treatment (*P* < 0.01) and time points (*P* < 0.01); the interaction between main effects was significant (*P* < 0.01). Scale bar = 100 μm. *P < 0.05, **P < 0.01, and ***P < 0.001.

**Figure 2:**
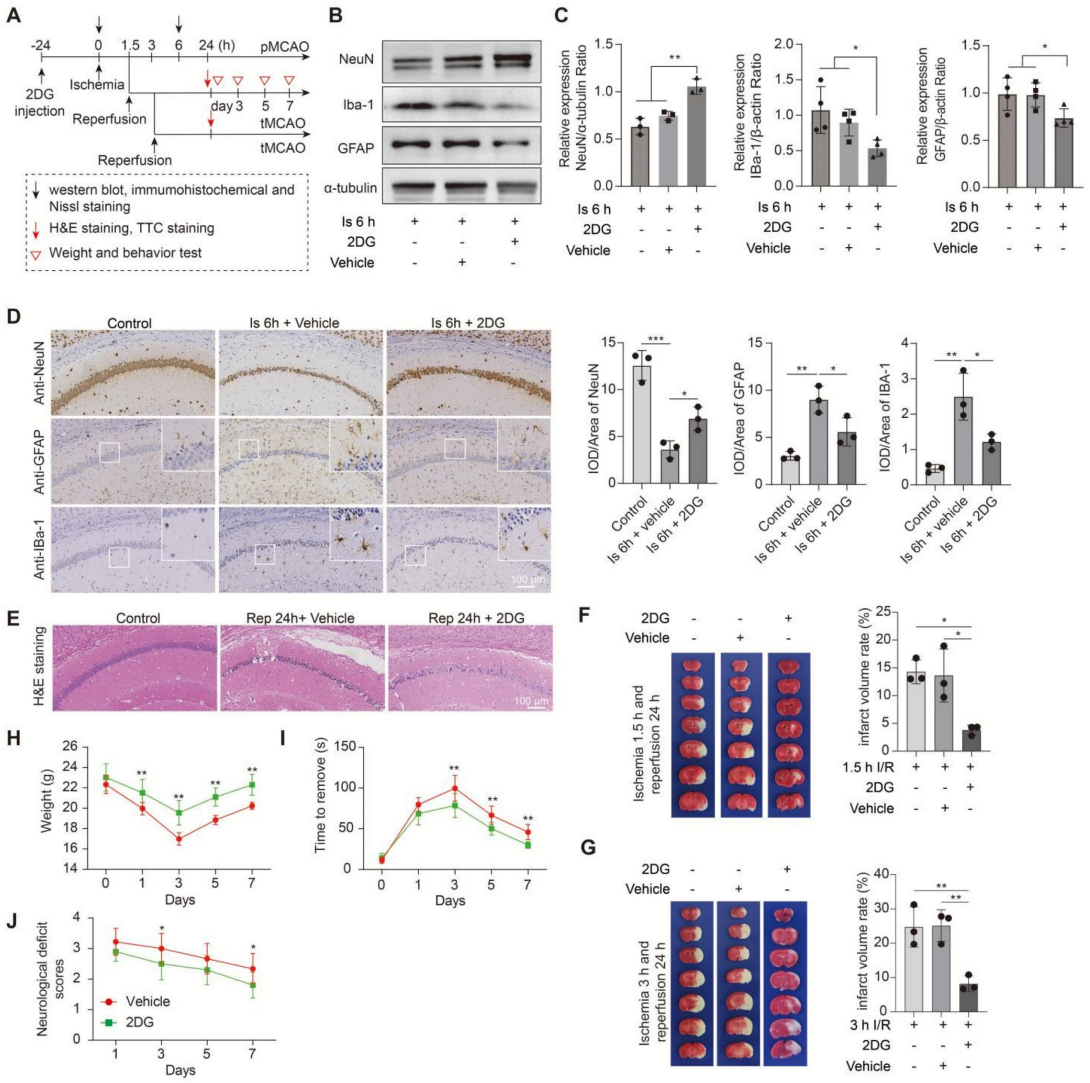
** Inhibition of the glycolytic pathway attenuates brain injury after ischemic stroke. A,** Experimental design and timeline of ischemic stroke, 2DG treatment, and analysis in mice. pMCAO and tMCAO indicate permanent and transient middle cerebral artery occlusion, respectively. **B-C,** The ischemic brain levels of neuroglial markers of NeuN, GFAP, and IBa-1 were measured in the tissues using western blotting at 6 h post cerebral ischemia after 2DG preconditioning (n = 3-4). **D,** Representative images and statistical analysis of immunohistochemical staining show the expression levels of neuroglial markers of NeuN, GFAP, and IBa-1 in the ischemic hippocampus CA1 region at 6 h post cerebral ischemia after 2DG preconditioning (n = 3). **E,** Representative images of hematoxylin and eosin (H&E) staining show damage in the ischemic hippocampus CA1 region at 24 h of reperfusion after 1.5 h of ischemia with 2DG preconditioning (n = 3). **F-G,** Representative images of 2,3,5-triphenyltetrazolium chloride (TTC) staining in brain sections at 24 h of reperfusion after 1.5 h (F) and 3 h (G) of ischemia. The infarction area is white (n = 3). **H-J,** Mice with the 2DG preconditioning treatment and ischemic stroke show better functional recovery in tests of body weight (H), sensory neglect (I), and neurological deficit scores (J. n = 12. Two-way ANOVA with repeated measures reported a significant effect of treatment (*P* < 0.01) and time points (*P* < 0.01); the interaction between main effects was significant (*P* < 0.01). Scale bar = 100 μm. *P < 0.05, **P < 0.01 and ***P < 0.001.

In this part experiments, we preconditioned the mice with Oxamate (a lactate dehydrogenase A [LDHA] inhibitor) via intraperitoneal injection and followed by the construction of middle cerebral artery occlusion (MCAO) model, then mainly observed the brain injury at two stages of ischemia and reperfusion, respectively, following the workflow (Figure 1A). No toxicity or death was observed in mice before MCAO surgery. In addition, the treatment of Oxamate has not much influence on the number of neurons, astrocytes, and microglial of normal mice when compared to the vehicle treated normal mice (Figure S1C). First, at the ischemia stage, we found that Oxamate preconditioning significantly reduced neuronal death, as evidenced by the results of western blot analysis (Figure 1B, C), immunohistochemical staining (Figure 1D and Figure S2A), and Nissl staining (Figure S2B). Similarly, the activated astrocyte and microglia were also reduced (Figure 1B-D and Figure S2A). Then, the hematoxylin and eosin (H&E) staining was performed and the results showed that brain edema and histological structure alterations of ischemia/reperfusion brains were alleviated when Oxamate preconditioning treatment was applied (Figure 1E and Figure S2C). Moreover, in addition to alleviating infarct volumes of ischemic brains with 90 min of ischemia and 24 h of reperfusion (Figure 1F), as well as in the ischemia 3h and reperfusion 24 h group (Figure 1G) and improved functional recovery (Figure 1H-J). Likewise, directly inhibiting the glycolytic pathway with 2-Deoxy-D-glucose (2DG; a glucose analogue that inhibits glycolytic pathway) preconditioning via intraperitoneal injection also phenocopied the beneficial effects of Oxamate (Figure 2 and Figure S3), corroborating previously published results 23 that 2DG pretreatment decreased neurological scores and reduced the infarct volume at 24 h after reperfusion in rats with ischemic stroke. These results suggested that inhibiting glycolytic pathway or lactate production during the ischemic stage shows neuroprotection for ischemic stroke.

### Increased brain lactate levels further aggravate brain injury after ischemic stroke

**Figure 3:**
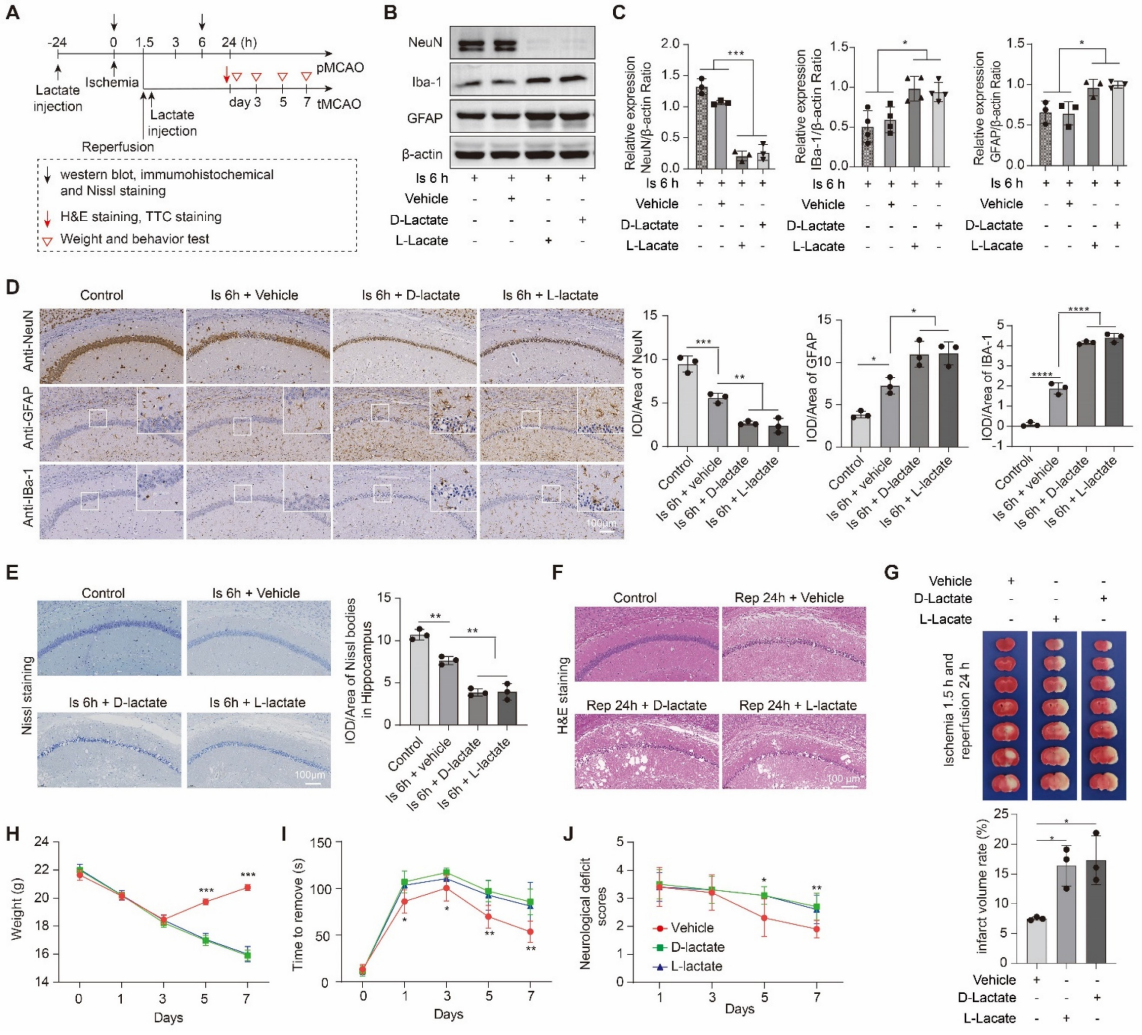
** Lactate supplementation before middle cerebral artery occlusion surgery exacerbates brain injury after ischemic stroke. A,** Experimental design and timeline of ischemic stroke, lactate treatment, and analysis in mice. pMCAO and tMCAO indicate permanent and transient middle cerebral artery occlusion, respectively. **B-C,** The ischemic brain levels of neuroglial markers of NeuN, GFAP, and IBa-1 were measured using western blot analysis of the tissues at 6 h after cerebral ischemia (n = 3-4). **D,** Representative images and statistical analysis of immunohistochemical staining show the expression levels of neuroglial markers of NeuN, GFAP, and IBa-1 in the ischemic hippocampus CA1 regions at 6 h after cerebral ischemia (n = 3). **E,** Representative images and statistical analysis of Nissl staining in the ischemic hippocampus CA1 regions at 6 h after cerebral ischemia (n = 3). **F,** Hematoxylin and eosin (H&E) staining shows the damage of the ischemic hippocampus CA1 regions after 24 h of reperfusion and 1.5 h of ischemia with lactate preconditioning (n = 3). **G,** Representative images of 2,3,5-triphenyltetrazolium chloride (TTC) staining of brain sections after 24 h of reperfusion and 1.5 h of ischemia. The infarction area is white (n = 3). **H-J** Mice with lactate preconditioning treatment and ischemic stroke show poorer functional recovery in tests of body weight (H), sensory neglect (I), and neurological deficit scores (J). n = 12. Two-way ANOVA with repeated measures reported a significant effect of treatment (*P* < 0.01) and time points (*P* < 0.01); the interaction between main effects was significant (*P* < 0.01). Scale bar = 100 μm. *P < 0.05, **P < 0.01, ***P < 0.001 and ****P < 0.0001.

To further confirm the effect of lactate accumulation on brain injury in ischemic stroke, we then further increased ischemic brain lactate levels by preconditioning treating the mice with sodium L-lactate and D-Lactate via intracerebroventricular (i.c.v.) injection. No toxicity or death was observed in mice before MCAO surgery. Additionally, the treatment of L-lactate also has not much influence on the number of neurons, astrocytes, and microglial of normal mice when compared to the vehicle treated normal mice (Figure S1C). As expected, in contrast to the results of reducing brain lactate levels, neuronal death and glial activation at the ischemia stage were markedly aggravated by preconditioning treatment with lactate (Figure 3A-E and Figure S4A, B). The brain edema and histological structure alterations of the ischemia/reperfusion brains (Figure 3F and Figure S4C) were significantly worsened by the lactate preconditioning treatment, which was consistent with the results of a previous report indicating that increased brain lactate is the key to the development of brain edema in rats with chronic liver disease 24. Furthermore, we found that the lactate preconditioning group had enlarged brain infarcts (Figure 3G) accompanied by poorer functional recovery, as shown by the results of the body weight test (Figure 3H), sensory neglect test (Figure 3I), and neurological deficit scores (Figure 3J). Therefore, these results strongly suggested that increased brain lactate levels caused by cerebral ischemia aggravate ischemic brain injury, resulting in poor outcomes of ischemic stroke.

### Increased brain lactate levels induce neuronal death and A1 astrocytes revealed by RNA-seq analysis

**Figure 4:**
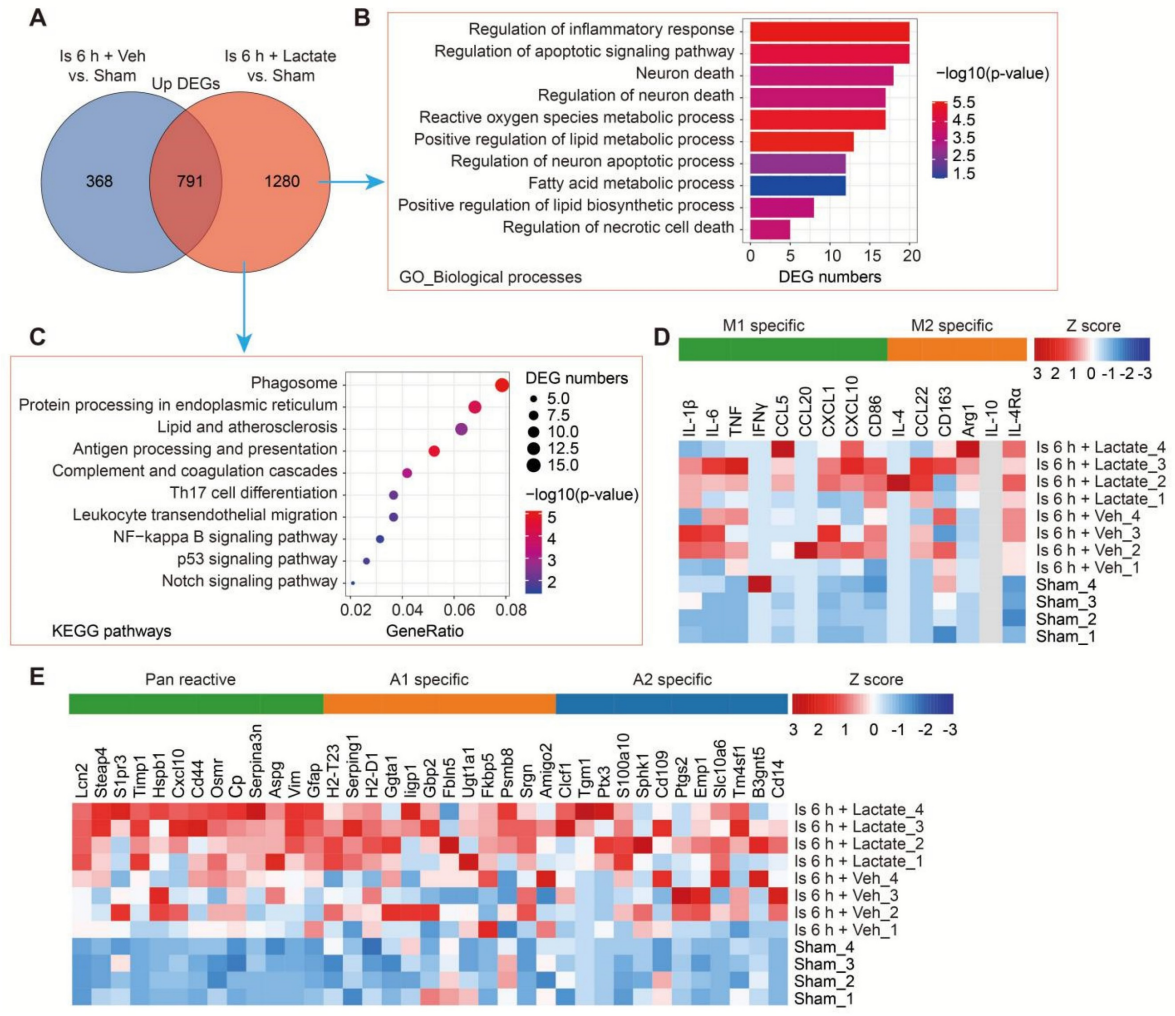
** Transcriptome profiles of ischemic brain tissues from mice with or without lactate preconditioning treatment. A,** Venn diagram showing overlapping and specifically upregulated differentially expressed genes (DEGs) of the lactate-treated and vehicle-treated ischemic brain tissues at 6 h after cerebral ischemia. **B-C** The Gene Ontology (GO) Biological Processes (B) and Kyoto Encyclopedia of Genes and Genomes (KEGG) (C) enrichment pathways of the lactate-treated specific upregulated DEGs. **D-E,** Heat map of reactive transcripts of microglia (D) and astrocytes (E) among sham, vehicle-treated, and lactate-treated ischemic mice. n = 4 for each group.

To examine how increased brain lactate levels during cerebral ischemia contribute to brain injury *in vivo*, we then performed RNA-seq to investigate the transcriptome profiles of ischemic brain tissues with or without preconditioning treatment of lactate via i.c.v. injection (Figure S5A). The RNA-seq data showed that the preconditioning treatment of lactate enlarged the numbers of differential expression genes (DEGs) when compared to the vehicle-treated ischemic brains (Figure S5B, C). Then, we mainly focused on the up-and down-regulated DEGs that were differentially regulated by additionally increased lactate in ischemic brain tissues, respectively. There are 1280 upregulated DEGs that were specifically identified in ischemic brain preconditioning treated with lactate (Figure 4A). GO-BP analysis showed that most of the upregulated DEGs were enriched in the pathways and during the processes of inflammation, apoptotic signaling pathway, neuron death, and metabolic process (Figure 4B). Phagosome, protein processing in endoplasmic reticulum, inflammation and immune response (e.g., antigen processing and presentation, complement and coagulation cascades, Th17 cell differentiation, Leukocyte transendothelial migration, and NF-kappa B signaling pathway), p53 signaling pathway, and Notch signaling pathway were also found as significantly enriched in the KEGG pathways among genes that were more upregulated in ischemic brain tissue preconditioning treated with lactate than in ischemic brains after vehicle preconditioning treatment (Figure 4C). Then, we found there are 740 specific down-regulated DEGs that were identified in ischemic brain preconditioning treated with lactate (Figure 4D). GO-BP analysis revealed that most of these DEGs were related to biological processes, such as regulation of synapse functions, Wnt signaling pathway, response to hypoxia, Notch signaling pathway, and positive regulation of neuron apoptotic process (Figure S5E), which was consistent with the results of the aggravation of neuronal death by lactate preconditioning treatment (Figure 3B-E and Figure S4A, B). In the KEGG analysis, we found that the Notch signaling pathway was enriched (Figure S5F), which has been demonstrated to be closely related to the fate of neurons after ischemic stroke 25.

To further assess glial reactivity in response to the increased lactate levels associated with ischemic stroke, we determined the gene expression changes in astrocytes 26 and microglial markers 27. As expected, both astrocytes and microglia exhibited normal responses in the sham mice but only with a significant induction of an A1 response (Figure 4E and Table S1) after cerebral ischemia, although there is an increase trend of M1 response (Figure 4D and Table S2). Furthermore, we found that a further induction of pan reactive and A1 specific markers of astrocytes in ischemic mice occurred as a result of lactate preconditioning (Figure 4E and Table S1), which was consistent with the results that reactive astrocytes were further ignited by lactate preconditioning treatment (Figure 3B-E and Figure S4A). While without much influence on the M1 microglia (Figure 4D and Table S2). These findings showed that reactive astrocytes are the main glial cells that respond to the increased brain lactate levels during the ischemia phase, which may be related to that the activated microglia released inflammatory factors promoting the transition of A1 astrocytes (Figure S5G) 26. These results of RNA-seq strongly suggested that the significant exacerbation of ischemic brain injury ascribed to increased brain lactate levels may be closely related to induced neuronal death and A1 astrocytes.

### Increased brain lactate levels caused by cerebral ischemia promote the formation of protein lactylation (Kla)

Next, we want to know how the increased brain levels at the ischemia stage contribute to the exacerbation of brain injury. Given PTMs are some of the most efficient biological mechanisms for regulating cellular pathophysiology 8, 9. Lactate has recently been shown to drive the formation of the protein Kla, which involves in the regulation of many pathophysiological processes. Therefore, we investigated whether protein Kla mediated the lactate-induced ischemic brain injury. The changes in protein Kla levels of the ischemic brain tissues (including ischemic cerebral cortex, hippocampus and striatum) were detected at different time points by performing western blot analyses with pan antibody. The results showed that protein Kla increased during the ischemia stage and reached a peak level at 6 h after ischemia (Figure S6A), which was consistent with the ischemic brain lactate levels after ischemic stroke (Figure S1B), indicating that increased brain lactate levels may drive the formation of protein Kla. While, the other PTMs (e.g., acetylation, succinylation) were not markedly changed in response to ischemic stroke (Figure S6B-G). These results strongly suggest that evidently increased protein Kla levels may participate in the contribution of brain injury caused by increased brain lactate levels after ischemic stroke.

**Figure 5:**
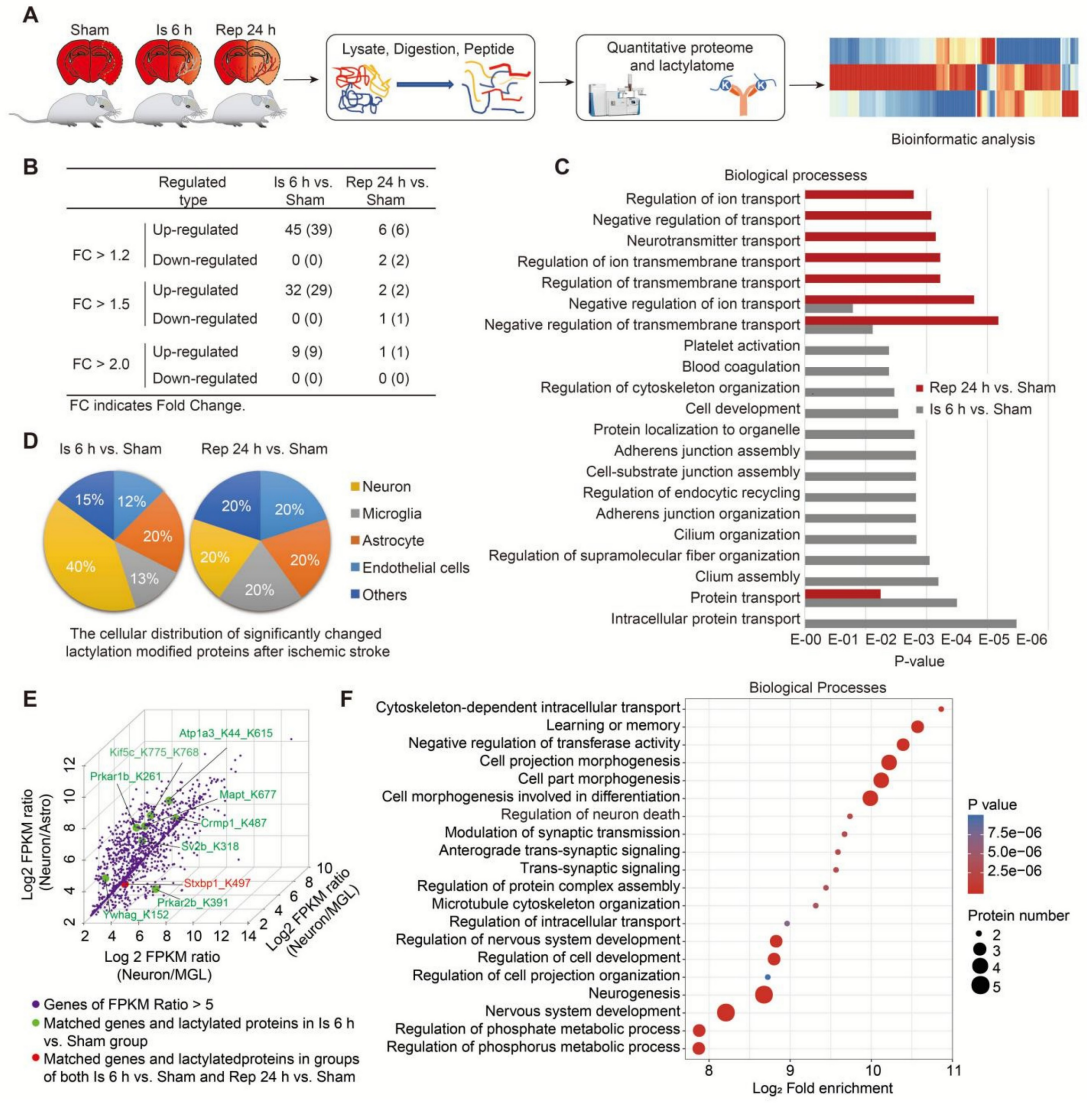
** Protein lactylation profiles of brains after ischemic stroke. A,** Schematic of the experimental strategy used to identify the proteome and lactylproteome changes in brains induced by cerebral ischemia and reperfusion. **B,** The number of significantly changed lactylations of proteins at different times. *P* < 0.05; Student's *t*-test. **C,** Gene Ontology (GO) biological process (BP) enrichment analysis of significantly different expressions of protein lactylation in brains with ischemia and reperfusion. *P* < 0.05; two-tailed Fisher's exact test. **D,** The pie charts show the major cellular distributions of significantly changed lactylation modifications of proteins induced by ischemia (right) and reperfusion (left). Significant changes in lactylated proteins (*P* < 0.05; Student's *t*-test) were matched with the relatively high-expression genes of neural cells (gene expression of one of the four neural cell types was higher than that of the others) from the published transcriptome data 28. **E,** Three-dimensional scatter plots show some significantly changed lactylated proteins that are mainly distributed into neurons. Significantly changed lactylated proteins (*P* < 0.05; Student's *t*-test) were matched with the relatively specific higher-expression genes of neural cells (gene expression of one of the four neural cell types was five-times higher than that of the other cellular types) from the published transcriptome data 28. **F** GO-BP enrichment analysis of the significantly changed lactylated proteins mainly distributed into neurons (E). n = 3 for each group.

Then, a label-free quantitative lactylproteomics analysis was performed to obtain a comprehensive understanding of the lactylproteome profiles of brain tissues of the sham, 6-h ischemia, and ischemia/reperfusion groups (Figure 5A and Figure S7). We found that all significantly changed protein Kla levels were upregulated after ischemia; however, only a few hyperlactylated or hypolactylated proteins were found in the ischemia/reperfusion brains (Figure 5B and Table S3), and the increase in lactyosites was independent of protein abundance (Figure S7B). Furthermore, the results of the Gene Ontology (GO) biological process (BP) enrichment analysis of differentially lactylated proteins showed that functional enrichments were markedly different between the ischemia and reperfusion (Figure 5C). For example, protein Kla during the ischemia phase were mainly enriched in the regulation of biological processes, such as cytoskeleton organization, regulation of endocytic recycling, and intracellular protein transport. In contrast, the regulation of ion and neurotransmitter transport mainly occurred during the reperfusion stage.

Next, to determine the cell type information (e.g., neurons, astrocytes, microglia, and endothelial cells) of significantly changed Kla on proteins after ischemic stroke, we performed a combined analysis of our lactylproteome data with previously published transcriptome data of RNA-seq of the mouse cerebral cortex 28 using we previously established method of conjoint analysis 29. Firstly, the relatively high expression genes of neural cells were defined as that the gene expression in one of the four neural cell types is higher than all the other three cellular types (ratio >1.0); when the ratio >5.0, it was defined as the relatively specific higher expression genes of neural cells. Then, we matched the relatively high-expression genes of neural cells (ratio >1.0) with the significantly changed lactylproteomes. The mRNAs matched by most of the significantly changed lactylated proteins were correlated with neurons during the ischemia stage (Figure 5D). Intriguingly, eight mRNAs matched with the significantly changed lactylated proteins were relatively high expression in neurons during the ischemia phase, and one was found in both the ischemia and reperfusion stages (Figure 5E). However, two were found in astrocytes during the ischemia and reperfusion stages, respectively (Atp1a2_K210la and Aldoc_K230la; Figure S7C). Additionally, only one was found in microglia during the ischemia phase (Edh1_K483, Figure S7D). Then, the GO-BP enrichment analysis of lactylated proteins with relatively specific high expression in neurons showed that these hyperlactylated proteins were mainly enriched in the regulation of neuronal functions, such as cell projection morphogenesis, cell part morphogenesis, cell morphogenesis involved in differentiation, regulation of neuron death, modulation of synaptic transmission, and anterograde trans-synaptic signaling (Figure 5F and Table S4), which was consistent with the results of RNA-seq (Figure 4B and Figure S5E). Among them, some of the lactylated proteins have been demonstrated to involve in regulating cell morphogenesis and differentiation. For example, Crmp1 is involved in the regulation of neurite outgrowth of murine cortical neurons, contributing to the neuronal morphogenesis 30-32 and is crucial for the mediation of neuronal differentiation 33. Kif5c is important for the cortical neuronal dendritic and spine growth and neuronal migration 34. Tau microtubule-associated proteins, encoded by the MAPT gene, are mainly expressed in neurons participating in axonal transport and synaptic plasticity 35, stabilizing axonal microtubules 36, and regulating the proliferation, migration and differentiation of Schwann cells 37. However, although the functional significance of Kla of these proteins in neuronal morphogenesis and differentiation after ischemic stroke remains largely unknown, the results suggested that increased protein Kla levels may be mostly enriched in neurons and participate in regulating neuronal biofunctions after cerebral ischemia.

Then, we further performed the immunofluorescent staining with pan antibody of protein Kla and found that the number of Kla+ cells was significantly increased after 6 h of cerebral ischemia (Figure S8A, B). Although the proportion of Kla+NeuN+ cells decreased significantly from about 82% in the sham group to about 73% in the ischemic group (Figure S8A, B), the number of Kla+NeuN+ cells was markedly increased in the ischemic cortex (Figure S8A, C). Additionally, the number of Kla+NeuN- cells was much lower than that of Kla+NeuN+ cells, but the proportion and number of Kla+NeuN- cells were both increased in the ischemic brains when compared to the sham groups (Figure S8B, C). The similar results were also found in the CA1, CA3 and DG regions of ischemic hippocampus (Figure S8D-L). These results suggested that most of the hyperlactylated proteins were mainly enriched in neurons during ischemia. Based on the findings, we proposed that neurons are the main neural cells where the protein Kla formation, and their activities may be influenced by protein Kla after ischemic stroke, especially during the ischemia stage.

### Astrocyte-derived lactate promotes protein lactylation after cerebral ischemia

**Figure 6:**
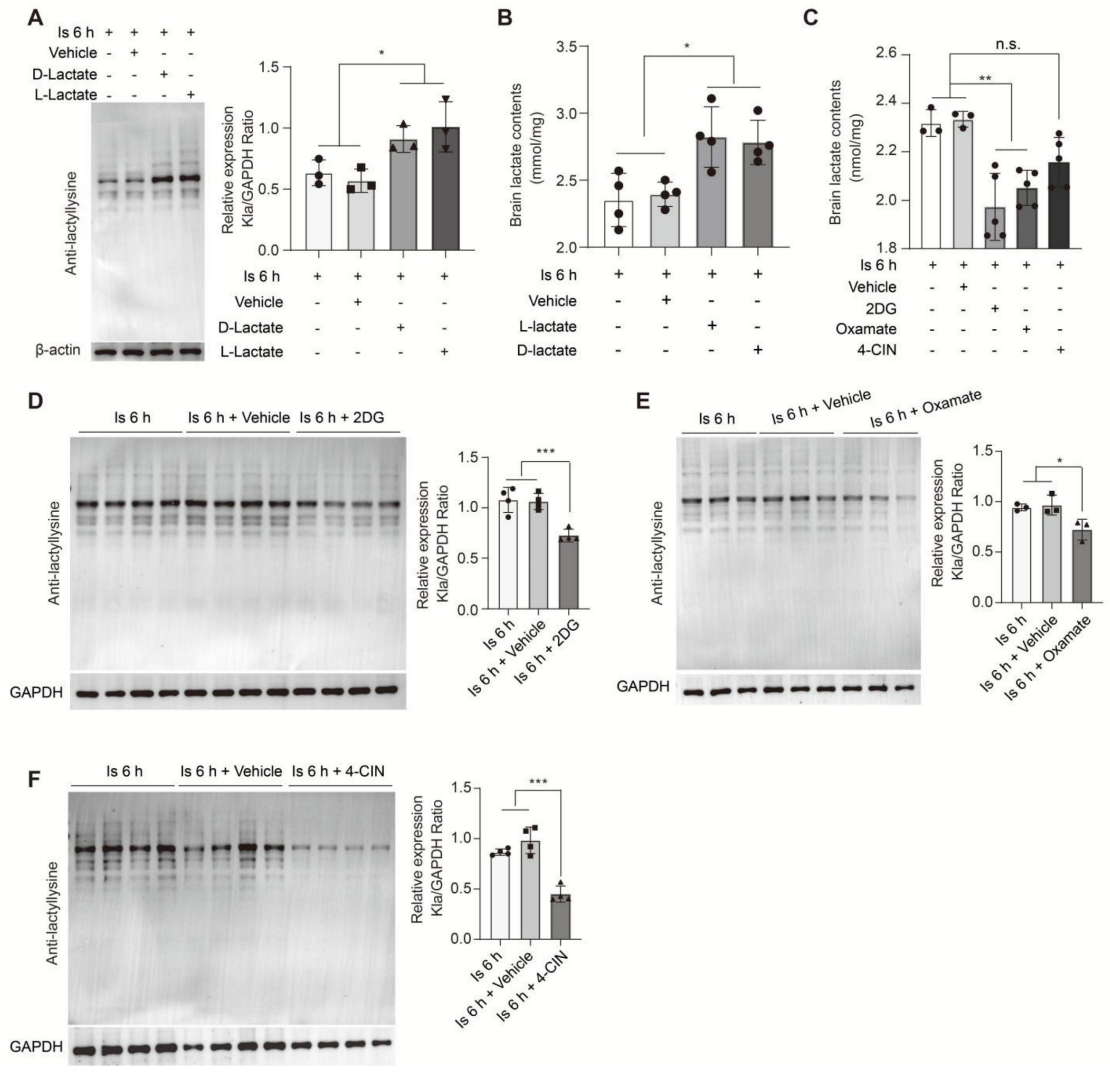
** Regulation of brain lactate levels significantly alters protein lactylation levels of ischemic brain tissue. A,** Preconditioning D-lactate and L-lactate intracerebroventricularly (i.c.v.) administered treatments increased the protein lactylation levels of ischemic brain tissues at 6 h after cerebral ischemia (n = 3). **B,** Lactate levels in the ischemic brain at 6 h after cerebral ischemia were measured after preconditioning D-lactate and L-lactate treatments administered via ICV injection (n = 4).** C,** Lactate levels in the ischemic brain at 6 h after cerebral ischemia were measured after preconditioning 2DG, oxamate, and 4-CIN treatments administered via intraperitoneal and ICV injection, respectively (n = 3-5). **D-F,** Preconditioning 2DG (D), Oxamate (E), and 4-CIN (F) treatments administered via intraperitoneal and ICV injection, respectively, markedly reduce the protein lactylation levels of ischemic brain tissues at 6 h after cerebral ischemia (n = 3-4). *P < 0.05, **P < 0.01; n.s. indicates a non-significant difference.

To further investigate whether the accumulation of lactate induced by cerebral ischemia would drive the formation of protein Kla, we performed supplementation of sodium L-lactate and D-lactate via i.c.v. injection. Subsequently, at the 6 h of cerebral ischemia, the brain levels of protein Kla were significantly increased in the L-lactate and D-lactate preconditioning groups compared to the vehicle group (Figure 6A), as well as the brain lactate levels (Figure 6B); while, protein Kla levels were not markedly changed in mice without experiencing cerebral ischemia (Figure S9A), suggesting that the accumulated lactate would drive the protein Kla formation mainly at the condition of cerebral ischemia insult. In contrast, lactate supplementation immediately after reperfusion did not influence the protein Kla levels of ischemia/reperfusion brains (Figure S9B). Then, to further confirm the results, we preconditioning delivered 2DG and Oxamate via intraperitoneal injection to inhibit lactate production. In this experiment, we found that the brain lactate levels were significantly decreased at 6 h of cerebral ischemia when preconditioning treated with 2DG and Oxamate, respectively (Figure 6C), while which had not much influence on the brain lactate levels of mice without experiencing cerebral ischemia (Figure S9C). As expected, the protein Kla levels were reduced (Figure 6D, E), but no changes occurred without ischemic insult (Figure S9A), suggesting that oxamate or 2DG pretreatment could obviously reduce the ischemic brain lactate and protein Kla levels although may not bring them to the levels comparable to those observed in sham mice. These results indicate that lactate accumulation after cerebral ischemia drives the formation of protein Kla.

**Figure 7:**
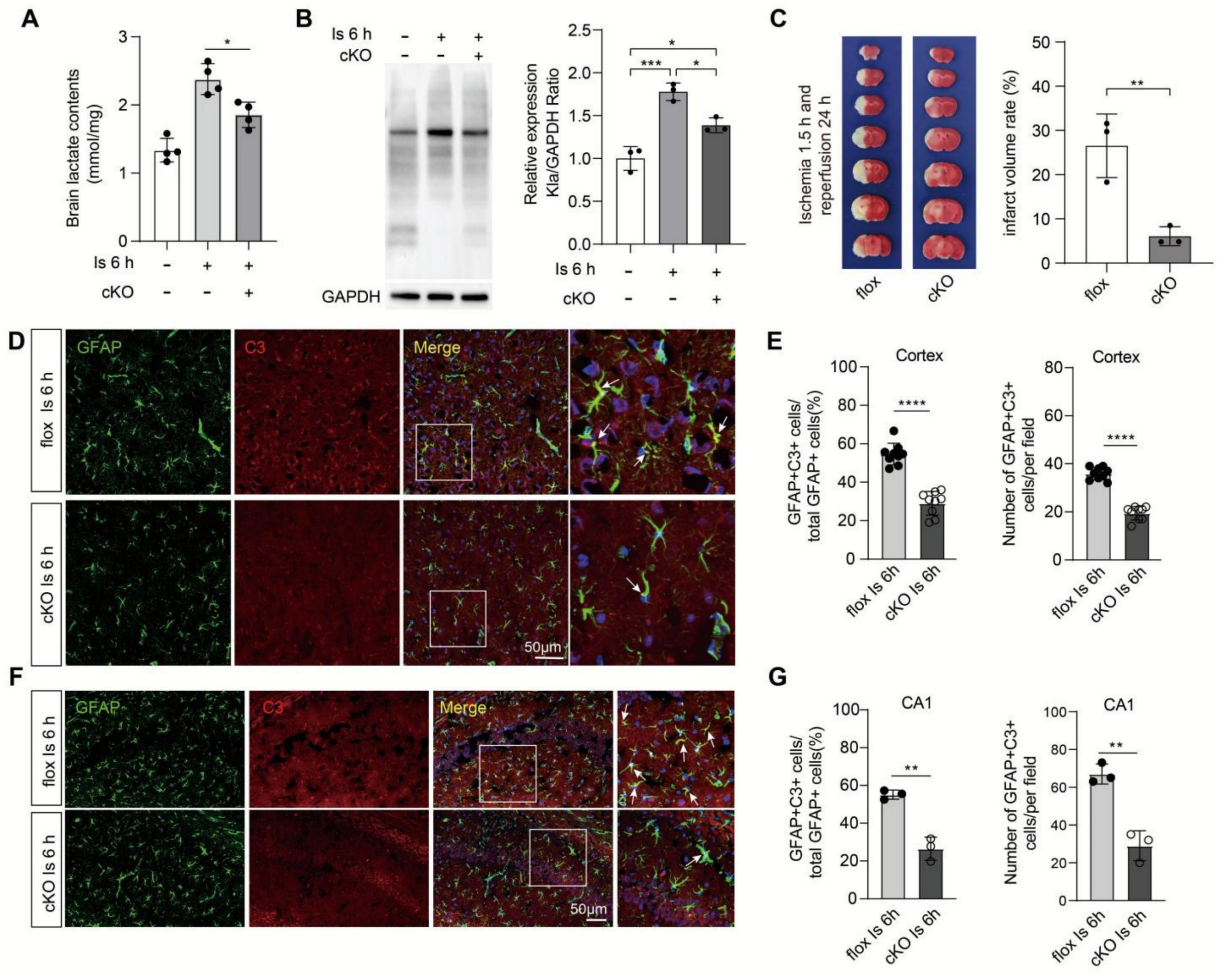
***Aldh1l1*^CreERT2^; *Ldha*^fl/fl^ mice showed better outcomes of ischemic stroke. A,** Lactate levels in the ischemic brain at 6 h were measured in cKO and flox mice (n = 4).** B,** *Aldh1l1*^CreERT2^; *Ldha*^fl/fl^ mice show decreased protein lactylation levels of ischemic brain tissues at 6 h after cerebral ischemia (n = 3). **C,** Representative images of 2,3,5-triphenyltetrazolium chloride (TTC) staining of brain sections at 1.5 h after ischemia and 24 h after reperfusion. The infarction area is white (n = 3). **D,** Representative image of colocalization staining with GFAP+ astrocytes (green) and C3 (red) in cortex of ischemic cKO mice compared to ischemic flox mice. **E,** Statistic analysis of the percentage of the GFAP+C3+/GFAP+ cells and number of GFAP+C3+ cells per field in cortex of ischemic cKO mice compared to ischemic flox mice. **F,** Representative image of colocalization staining with GFAP+ astrocytes (green) and C3 (red) in CA1 region of hippocampus of ischemic cKO mice compared to ischemic flox mice. **G,** Statistic analysis of the percentage of the GFAP+C3+/GFAP+ cells and number of GFAP+C3+ cells per field in CA1 region of hippocampus of ischemic cKO mice compared to ischemic flox mice. Scale bar = 100 μm for the (C) upper and 20 μm for the (C) lower. **P < 0.01.

Due to their lower glycolytic capacity, neurons mainly uptake lactate from extracellular sources via the monocarboxylate transporter 2 (MCT2), a high-affinity MCT present in neurons 1. Then, the i.c.v injection of the MCT2 inhibitor α-cyano-4-hydroxycinnamate (4-CIN) 38, 39 was performed to investigate whether the markedly increased neuronal protein Kla levels would be prevented by blocking the lactate shuttle to neurons. We found that the protein Kla levels of ischemic brains preconditioned with 4-CIN treatment were also significantly decreased (Figure 6F) but with little influence on lactate production (Figure 6C). In addition, it is interesting that the relatively large glycolytic capacity of brain tissue is most likely attributed to astrocytes 40, 41, and increasing evidence shows that the astrocyte-neuron lactate shuttle is the main way by which lactate transfers to neurons to meet the neuronal energy needs 42. Accordingly, we constructed astrocyte conditional Ldha knockout [Aldh1l1^Cre^; Ldha^fl/fl^ (cKO)] mice by crossing the Aldh1l1^Cre^ mice with Ldha^fl/fl^ mice (Figure S10A-C). Then, we found that the cKO mice that experienced ischemic injury showed decreased brain lactate levels (Figure 7A), protein Kla levels (Figure 7B), and infarct volumes than the control mice (Figure 7C). In addition, we also found that the expression of A1 marker C3 in cKO mice with cerebral ischemia was decreased in both cortex and hippocampus when compared to the control mice (Figure 7D-G and Figure S10D-G). Therefore, these results strongly suggest that increased neuronal levels of protein Kla after cerebral ischemia was mainly derived from astrocytic lactate, which promotes the brain injury of ischemic stroke.

### Pharmacological intervention for the formation of protein lactylation offers neuroprotection for ischemic stroke

To date, p300 has been shown as the sole Kla writer protein 12 that drives the formation of protein lactylation. Then, we investigated whether directly inhibiting lactylation formation via targeting p300 could also reduce the formation of protein Kla and provide therapeutic effects for ischemic stroke. First, we examined the expression of p300 in the ischemic brains using the qPCR and the results showed there was an increasing trend in the expression of p300 in the ischemic brains but with little significance between sham and ischemia groups (Figure S11A), suggesting that the increase in the formation of protein Kla may be ascribed to the increase in lactate. In addition, class I histone deacetylases (HDAC1-3) have been shown as the histone lysine delactylases 43. Then, we detected their expressions and found that the expression of HDAC1 and 2 were not markedly changed in ischemic brains when compared to sham groups, while the expression of HDAC3 was markedly downregulated in response to the ischemic brain injury (Figure S11B), suggesting that downregulation of HDAC3 may be related to the formation of Kla.

**Figure 8:**
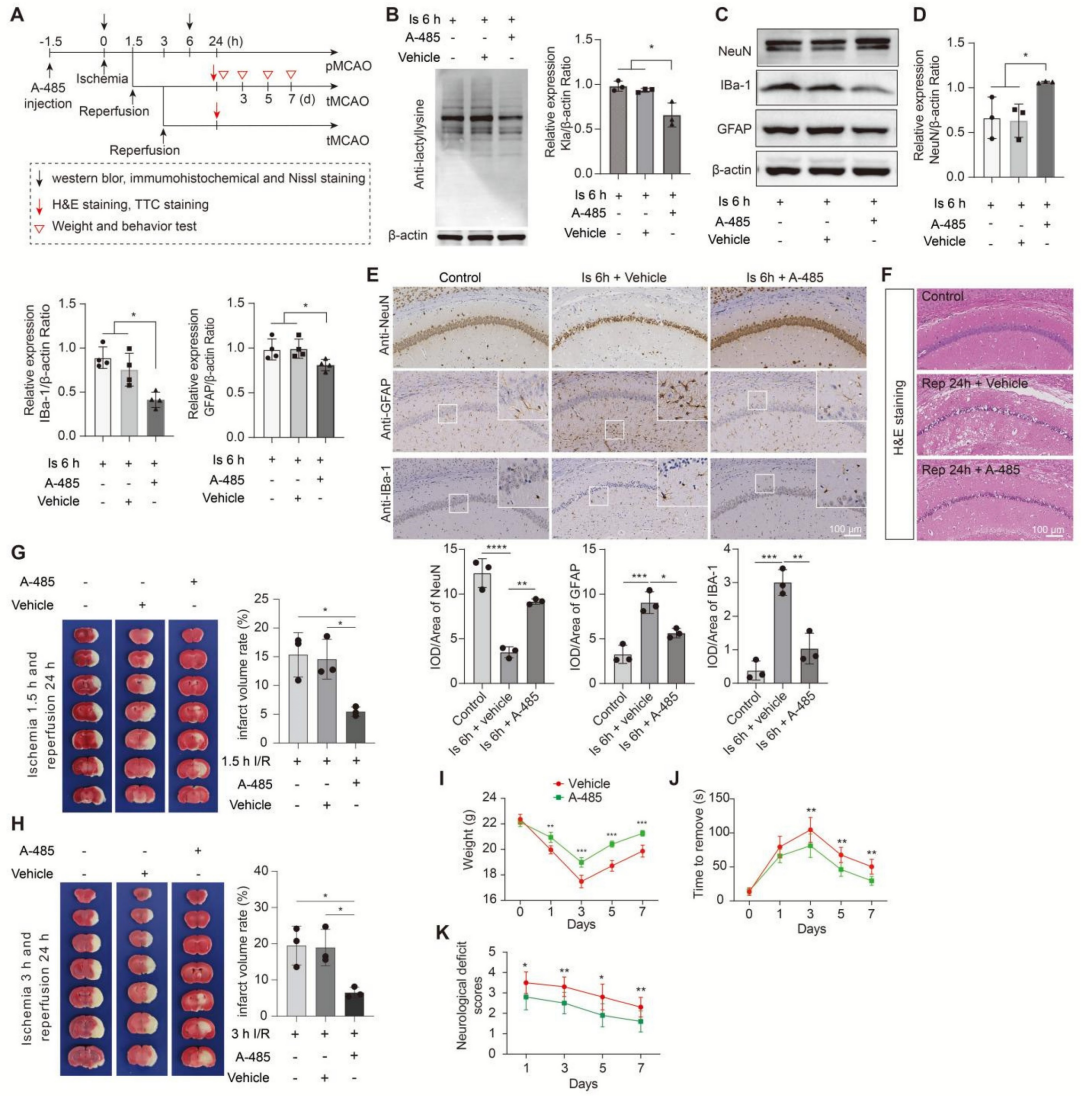
** Inhibition of the formation of protein lactylation attenuates brain injury after ischemic stroke. A,** Experimental design and timeline of ischemic stroke, A-485 treatment, and analysis in mice.** B,** Representative images of western blot analysis show whole protein lactylation levels of ischemic brain tissues at 6 h after cerebral ischemia (n = 3).** C-D,** The ischemic brain levels of neuroglial markers of NeuN, GFAP, and IBa-1 were measured using western blot analysis of tissues at 6 h after cerebral ischemia and A-485 preconditioning (n = 3-4). **E,** Representative images and statistical analysis of immunohistochemical staining show the expression levels of neuroglial markers of NeuN, GFAP, and IBa-1 in the ischemic hippocampus CA1 regions at 6 h after cerebral ischemia and A-485 preconditioning (n = 3). **F,** Hematoxylin and eosin (H&E) staining show damage of the ischemic hippocampus CA1 regions at 24 h after reperfusion and 1.5 h of ischemia with A-485 preconditioning (n = 3). **G-H,** Representative images of 2,3,5-triphenyltetrazolium chloride (TTC) staining of brain sections at 24 h after reperfusion and 1.5 h (G) and 3 h (H) after ischemia. The infarction area is white (n = 3). **I-K** Mice treated with A-485 preconditioning and ischemic stroke show better functional recovery in tests of body weight (I), sensory neglect (J), and neurological deficit scores (K). n = 12. Two-way ANOVA with repeated measures reported a significant effect of treatment (*P* < 0.01), the interaction between main effects was also significant (*P* < 0.01). Scale bar = 100 μm. *P < 0.05, **P < 0.01, ***P < 0.001 and ****P < 0.0001.

Given that HDAC3 inhibition has been shown to be neuroprotection for ischemic stroke with non-delactylase functions 44-47, suggesting that there may exist complex regulative mechanisms that deserve further deep investigation. Then, we only chose A-485, a potent and specific small molecule inhibitor of p300 48, 49, to treat mice before MCAO surgery in this study. No toxicity or accidental death was also observed in mice. As expected, A-485 preconditioning treatment significantly reduced protein Kla levels in 6 h ischemia brains (Figure 8A, B) without influencing the lactate production (Fig. S11C) and other PTMs levels, such as acetylation, succinylation, etc. (Fig. S11D-I). Likewise, A-485 treatment showed neuroprotection evidenced by improved neuronal survival (Figure 8C-E and Figure S12A, B), decreased glial activation (Figure 8C-E and Figure S12B), and alleviated brain edema and histological structure alterations of the ischemic brain (Figure 8F and Figure S12C). In addition, A-485 reduced the infarct volumes of the ischemic brain (Figure 8G), extended the reperfusion time (Figure 8H), and improved the functional recovery of mice with ischemic stroke (Figure 8I-K). These results further indicate that the increased brain lactate levels aggravate brain injury after ischemic stroke may be largely related to driving the formation of protein Kla, which can be a therapeutic target that reduces brain injury and extends the time window of ischemic stroke treatment.

## Discussion

Ischemic stroke is a type of acute metabolic disorder, especially during the ischemia phase, and the degree of injury greatly influences the outcomes of recanalization treatment. However, the damage mechanisms of the ischemia phase were still largely unknown, especially for the metabolism-related mechanisms. Many studies have found that increased brain lactate levels were mainly at the ischemia stage, but its role in regulating ischemic brain injury remains exploration. Although studies have shown that lactate supplementation at the reperfusion stage of ischemic stroke offers neuroprotection in mice, whether their roles at the ischemia stage were similar to the reperfusion stage as lactate usage as neuronal fuel needs normal oxygen tension. Therefore, in this study, we first investigate the roles of increased brain lactate at the ischemia stage in influencing brain injury of mice with ischemic stroke and found that astrocytic lactate aggravates the ischemic brain injury via driving the formation of protein Kla. Our study provides new insights into understanding the role and mechanism of lactate at the ischemia stage in contributing to the pathological processes of brain injury of ischemic stroke in mice and may offer a novel therapeutic target for the treatment of ischemic stroke.

In addition to that lactate in the brain is formed predominantly in astrocytes and transferred to neurons to meet the neuronal energetic requirements for supporting neuronal activity under physiological conditions 1, neurons have also been demonstrated to possess the ability to maintain their normal function by metabolizing glucose through glycolysis to supply tricarboxylic acid (TCA) cycle metabolites by a recent study in mice 50. However, due to little energy material reserve in brain except a certain amount of glycogen was stored in astrocytes 2, and high energy consumption for neuronal activity. Therefore, once the cerebral ischemia happens, astrocytic lactate may become the main source of energy materials for neurons. Additionally, the loss of normal oxygen tension after cerebral ischemia penalize neuronal mitochondrial lactate oxidization to produce ATP, resulting in the lactate accumulation in neurons and then promoting the formation of protein Kla, theoretically. Thus, in our study, we found that astrocytic lactate increased protein Kla levels in mouse brains after cerebral ischemia and that was mainly located in neurons.

In addition to fueling neurons, lactate has been found to have some novel roles. However, the functions of lactate have been a subject of some debate on neurogenesis, with previous studies showing that it can both hinder 5 and support 51 adult mouse hippocampal neurogenesis; different dosages and administration approaches may be explanations for this debate. In addition, experimental evidence has suggested that lactate also strongly affects neuronal excitability 52. Regarding ischemic stroke, although studies showed that L-lactate supplement offered neuroprotection against brain injury of animal models 7, 53, 54, as well as the D-lactate intravenous administration 7, in this study, we found that both L-lactate and D-lactate markedly aggravated brain injury caused by ischemic stroke of mice. Although, theoretically, the increased levels of ischemic brain lactate can be used as an energy substrate for neurons; at the ischemia phase, the absence of the partial pressure of oxygen (pO2) impairs the oxidative capacity of the brain 1. Therefore, the accumulation of lactate at the ischemia phase cannot be metabolized for energy production but drove the formation of protein Kla in the ischemic brains that were found in this study. In contrast, during the reperfusion stage with normal oxygen tension, lactate can be used as neuronal fuel and offer neuroprotection for mice with ischemic stroke 7. Accordingly, we did not detect an increase in the protein Kla levels in the normal brains even additional supplemented with lactate and in ischemic/reperfusion brains supplemented lactate at the reperfusion stage in this study, but the latter markedly reduced the cerebral infarct of ischemic stroke. Together, these results strongly suggest that lactate has multiple functions during pathophysiological processes of brain diseases and whether lack of oxygen would determine the lactate fate for neuroprotection or neurotoxic in the biological processes of ischemic stroke. Collectively, current researches showed lactate possesses complex roles in regulating biological processes. However, although lactate has been shown to be detrimental or neuroprotection at different stages of ischemic stroke, whether the other roles of lactate, like emerging signaling roles, also participate in the regulation of ischemia and reperfusion injury remains elusive in future.

PTMs have important roles in regulating diverse pathophysiological processes and pathways by influencing protein structures and functions. Kla, a newly discovered PTM, has been found to regulate gene expression in macrophages by regulating histone modification 12. Several studies have investigated the roles of histone Kla in regulating inflammation 55, lung myofibroblasts 14, and cancer 56, 57 of animal models. In addition, one study used the LC-MS/MS to perform a global lysine lactylome analysis of the plant fungal pathogen *Botrytis cinerea* and provided some basic information regarding non-histone protein Kla 58. These results suggest that the non-histone protein Kla could participate in many biological processes of animal diseases models. In this study, we demonstrated that the non-histone protein Kla contributes to brain injury after cerebral ischemia in mice. The bioinformatic analysis showed that protein Kla is mainly distributed in neurons that was further confirmed by the results of immunofluorescent staining, whereas pharmacological inhibition of lactate transfer to neurons via MCT2 significantly reduced protein Kla levels. These results indicate that increased protein Kla levels in neurons may be correlated to their death. Additionally, we found that the induction of A1 astrocytes during the ischemia stage may be closely related to protein Kla as energy supply is the basis of cellular activity. Because as a glycolytic enzyme, aldolase C (Aldoc) is mainly expressed in astrocytes 28, suggesting that the increase of Aldoc_K230la might promote glycolysis to provide the energy for astrocyte activity that needs to be further investigated. Additionally, RNA-seq data showed that the transcriptive levels of brain inflammatory cytokines (e.g., IL1α, C1q) that strongly induce A1 astrocytes have been further obviously upregulated when lactate was preconditioning treated. Furthermore, targeting the formation of protein Kla also showed a significant reduction in the activation of glial cells. Additionally, directly reducing the astrocytic lactate production using the *Aldh1l1*^CreERT2^; *Ldha*^fl/fl^ mice, we found that astrocytic lactate promoted the formation of protein Kla and resulted in poor outcomes of mice with ischemic stroke. Therefore, astrocytic lactate-derived protein Kla exacerbates neuronal death and activation of glial cells, resulting in exacerbation of brain injury in mice with ischemic stroke, especially at the ischemia stage.

However, in this study, there are some limitations. First, the detailed roles and mechanisms of how enhanced protein Kla contribute to neuronal death and glial activation still need to be investigated in the future. Second, the protein Kla writer, p300, is not specific. Because p300 is primarily found as the writer of acetylation 59, 60. Although we found that the p300 inhibitor A-485 did obviously reduce the formation of protein Kla but not significantly influenced the protein acetylation levels and other PTMs levels after ischemic stroke, exploring more specific protein Kla writer(s) in future studies will be more conducive to study the roles and mechanisms of protein Kla in regulating pathophysiological processes of relative diseases. Finally, given that no effective technologies currently can be applied to directly inhibit glycolytic pathway in the ischemic brain tissues immediately after cerebral ischemia happens, therefore, we chose to administrate drugs in a preconditioning way to enrich as much as the drugs in the brain before ischemia happens so that can immediately exert inhibiting roles but without influence much to the normal brain metabolism. Therefore, developing a method in future that can accurately deliver drugs to ischemic tissue simultaneously with MCAO surgery will overcome this issue.

In conclusion, our findings shed new light on the mechanism underlying the key contribution of astrocytic lactate-derived protein Kla in the development of ischemic brain injury in mice. Protein Kla is an attractive therapeutic target that can reduce ischemic neuronal injury, extend the time window for ischemic stroke treatment, and offer neuroprotection from ischemic stroke. By studying ischemic injury within the time window for ischemic stroke in mice, we may provide a novel potential therapeutic strategy for the comprehensive treatment of patients with ischemic stroke.

## Methods

### Mice

C57BL/6 male mice 8 weeks of age (weight, 23 ± 2 g) purchased from Gempharmatech (Chengdu, China) were used in this study. The mice were housed in a specific pathogen-free environment with a 12-h/12-h light/dark cycle, room temperature of 25°C, and relative humidity of 45-55%. They had free access to food and water. All animal protocols used in this study were approved by the Animal Care and Use Committee and the Institutional Animal Care and Use Committee of the Institute of Model Animals of Chengdu University of Traditional Chinese Medicine and the Animal Ethics Committee of Army Medical University (Third Military Medical University). All experiments were performed according to the Guide for the Care and Use of Laboratory Animals published by the National Academy of Sciences and the National Institutes of Health. No abuse or maltreatment occurred during our study.

### Generation of *Ldha*^flox/flox^ × *Aldh1l1*^CreERT2^ bigenic mice

To generate bigenic mice, *Ldha*^flox/flox^ (*Ldha*^fl/fl^) mice were crossed with hemizygous *Aldh1l1*^CreERT2^ mice 61. Bigenic pups were identified by genotyping for* loxP* sites (forward primer 5'-CTG AGC ACA CCC ATG TGA GA-3'; reverse primer 5'-AGC AAC ACT CCA AGT CAG GA-3') and Cre/ERT2 (forward primer 5'-GGC AAA CGG ACA GAA GCA-3'; reverse primer 5'-CTT CAA CAG GTG CCT TCC A-3') in two separate PCR reactions for each pup. Bigenic mice used for experiments were homozygote for *loxP* sites flanking exon 3 of the *Ldha* gene and hemizygous for the *Aldh1l1*^CreERT2^ insert (*Ldha*^fl/fl^; *Aldh1l1*^CreERT2^). These mice were purchased from the Jackson Laboratory (Sacramento, CA, USA): JAX Stock No. 030112: B6(Cg)-*Ldha*^tm1c(EUCOMM)Wtsi^/DatsJ with a common name of “*Ldha*^flox^,” and JAX Stock No. 031008: B6N.FVB-Tg (*Aldh1l1*-cre/ERT2)1Khakh/J with a common name of “*Aldh1l1*-Cre/ERT2 BAC transgenic (C57BL/6N).”

### Tamoxifen administration

Mice with *Ldha* specific knockout in astrocytes were induced by tamoxifen (Sigma, T5648). As previously described 62, tamoxifen was freshly prepared at a final concentration of 20 mg/ml in corn oil and dissolved overnight with continuous agitation. 6-week-old of each group mice were administered intraperitoneally with 100 μl tamoxifen per mouse for five consecutive days. Experiments were performed two weeks after the last tamoxifen injection.

### Middle cerebral artery occlusion models

The mouse stroke models were established by occluding the left middle cerebral artery (MCA) as performed in our previous studies 63. After mice were anesthetized with pentobarbital sodium (50 mg/kg), the left common, internal, and external carotid arteries were exposed. Then, a 7-0 silicon-coated monofilament nylon suture (MSMC21B120PK50; RWD Life Science, China) was inserted in the internal carotid artery and blocked the origin of the MCA. The filament was withdrawn at 1.5, 3, 6, 12, and 24 h to establish transient MCA occlusion (MCAO) models; after 1.5 h and 3 h ischemia, the filament was withdrawn, and the suture knot around common carotid arteries was loosened to induce the cerebral ischemia and reperfusion. The regional cerebral blood flow during surgery and after surgery was monitored by a laser speckle imaging system (RFLSI Pro^+^; RWD Life Science, China). Unsuccessful MCAO models, including asymptomatic and dead mice before euthanasia, were excluded from this study.

### 2,3,5-triphenyltetrazolium chloride staining

Mice were euthanized and their brains were collected after 24 h of reperfusion for 2,3,5-triphenyltetrazolium chloride (TTC) histology (93145; Sigma Aldrich, St. Louis, MO, USA). The animals were euthanized and their brains were extracted and immediately frozen at -20°C for 20 min. During this time, TTC (1% in phosphate-buffered saline) was freshly prepared. The brains were sectioned coronally to 1-mm-thick slices and stained with 1% TTC at 37°C for 30 min. The slices were turned over every 15 min. Then, the brain slices were placed in 4% paraformaldehyde (PFA) for 1 h. Normal brain tissue stained brightly, and the infracted areas were pale white.

### Measurement of ischemic brain levels of lactate

The ischemic brain tissues obtained after 1.5, 3, 6, 12, and 24 h of cerebral ischemia and tissues obtained after 1.5 h of ischemia and 24 h of reperfusion were prepared for the lactate assay. In addition, the brain tissues of ischemic mice pretreated with 2DG, oxamate, A-485, or 4-CIN obtained at 6 h after cerebral ischemia were also used for lactate measurements. The ischemic brain lactate concentrations were measured using a lactate assay kit (MAK064; Sigma Aldrich) and assayed according to the manufacturer's instructions.

### Immunohistochemistry

Based on our previously reported method 64, we performed immunohistochemical staining of the brain samples. Briefly, at 6 h after cerebral ischemia, appropriate numbers of control mice and lactate-treated, 2DG-treated, Oxamate-treated, or A-485-treated mice were perfused with 4% PFA (Sigma Aldrich) in phosphate-buffered saline for 15 min to fix their brains. Then, the brains were post-fixed for 48 h in 4% PFA 4% at 4°C. After cryoprotection in a sucrose gradient, 4-μm-thick sections were cut. According to the standard protocol, paraffinizing, dewaxing, dehydrating, antigen retrieval, and blocking were performed sequentially. Incubation with primary antibodies GFAP (1:1000; ab53554; Abcam), Iba1 (1:1000; ab5076; Abcam), and NeuN (1:1000; ab177847; Abcam) was performed at 4°C overnight. Then, washing, blocking, and incubation with secondary antibodies (1:200; A11008 and A11055; Thermo Scientific, Waltham, MA, USA) and chromogen development using DAB (1:50; G1211; Servicebio, Wuhan, China) were performed. Three different brain regions of cortex and striatum from three brain sections per animal were analyzed, and the CA1 region of hippocampus from three brain sections per animal were analyzed in our study. The samples were observed under a Nikon Eclipse E100 microscope with a 20× objective (DS-U3; Nikon, Tokyo, Japan). The statistical results were shown as “IOD/Area”. IPP 6.0 image processing software was used to count the IOD of NeuN-, IBA-1-, and GFAP-positive cells.

### Hematoxylin and eosin staining

According to our previously reported method 65, we performed hematoxylin and eosin (H&E) staining. The brain tissues obtained at 24 h after reperfusion were fixed with buffered 4% PFA for 48 h. All samples were cut into 5-μm sections. Then, the sections were stained with H&E. Three different brain regions of cortex and striatum from three brain sections per animal were analyzed, and the CA1 region of hippocampus from three brain sections per animal were analyzed in our study. These brain slices were observed under a Nikon Eclipse E100 microscope with a 20× objective (DS-U3; Nikon).

### Nissl staining

We performed Nissl staining according to our previously reported method 65. Briefly, the brains were collected at 6 h after cerebral ischemia for Nissl staining to assess neuronal survival. The 4-μm brain sections were stained with cresyl violet and hydrated in 1% toluidine blue for 40 min. Then, they were washed with deionized water and differentiated with 70% alcohol for 5 min. Three different brain regions of cortex and striatum from three brain sections per animal were analyzed, and the CA1 region of hippocampus from three brain sections per animal were analyzed in our study. Samples were observed using a Nikon Eclipse E100 microscope with a 20× objective (DS-U3; Nikon). The statistical results were shown as “IOD/Area”. IPP 6.0 image processing software was used to count the IOD of Nissl bodies.

### Western blotting

The ischemic brain tissue extracts were prepared using RIPA lysis buffer (FMS-WB035) and centrifuged at 13,000 rpm for 10 min at 4°C. The soluble protein solutions were mixed with 5× loading buffer (Beyotime, Shanghai, China) and heated at 95°C for 7 min. Equal amounts of protein (15 μg) from each sample were separated using 10% Tris-Gly PAGE and transferred to polyvinylidene difluoride membranes (Merck & Millipore, Burlington, MA, USA). After blocking with 5% skim milk for 1.5 h at room temperature, the membranes were incubated with antibodies (pan anti-lactyllysine antibody [PTM-1401; 1:1000 dilution; PTM Bio, Zhejiang, China], pan anti-lactyllysine (Kla) antibody [PTM-1401RM; 1:1000 dilution; PTM Bio], pan anti-succinyllysine antibody [PTM-401; 1:1000 dilution; PTM Bio], pan anti-malonylysine antibody [PTM-901; 1:1000 dilution; PTM Bio], pan anti-2-hydroxyisobutyryllysine antibody [PTM-801; 1:1000 dilution; PTM Bio], pan anti-β-hydroxybutyryllysine antibody [PTM-1201; 1:1000 dilution; PTM Bio], pan anti-crotonyllysine antibody [PTM-501; 1:1000 dilution; PTM Bio], pan anti-acetyllysine antibody [PTM-101; 1:1000 dilution; PTM Bio], goat anti-GFAP antibody (ab53554; 1:1000 dilution; Abcam, Cambridge, UK], rabbit anti-NeuN antibody [ab177487; 1:1000 dilution; Abcam], goat polyclonal anti-Iba1 antibody [PA5-18039; 1:1000 dilution; Thermo Fisher], rabbit monoclonal anti-HDAC3 antibody [ab32369; 1:1000 dilution; Abcam], rabbit monoclonal anti-HDAC2 antibody [ab32117; 1:1000 dilution; Abcam], rabbit monoclonal anti-HDAC1 antibody [PTM-6350; 1:1000dilution; PTM Bio], mouse monoclonal anti-β-actin [ZB15001; 1:1000 dilution; Servicebio], and anti-GAPDH [60004; 1:1000 dilution; Proteintech, Rosemont, IL, USA]) overnight at 4°C. The membranes were washed three times in Tris buffered with Tween-20 and then incubated with the secondary antibody (1:3000; Proteintech) at room temperature for 1.5 h. Immunoblotting signals were visualized using the ECL Kit (Thermo Scientific) and an imaging system (Imager 600; Amersham, Amersham, UK).

### HPLC-MS/MS analysis

The ischemic brain samples obtained from sham mice, from mice after 6 h of cerebral ischemia, and from mice after 1.5 h of ischemia and 24 h of reperfusion were used for the high-performance liquid chromatography with tandem mass spectrometry (HPLC-MS/MS) analysis. The protein concentration of the supernatant was determined using the bicinchoninic acid method. Then, the protein samples were digested with trypsin. After pan anti-lactyllysine antibody (PTM-1401, lot: Z264J028P0; PTM Bio) was conjugated to Protein A Sepharose beads, the tryptic peptides dissolved in NETN buffer (100 mM NaCl, 1 mM EDTA, 50 mM Tris-HCl, 0.5% NP-40, pH 8.0) were incubated with pre-washed pan anti-lactyllysine antibody beads at 4°C overnight with gentle shaking. The tryptic peptides were dissolved in buffer A (0.1% formic acid in water, v/v) and loaded onto a homemade reverse-phase analytical column (length: 15 cm; inner diameter: 75 μm; particle size: 3 µm) connected to an EASY-nLC 1000 UPLC system (Thermo Fisher Scientific). The gradient of HPLC buffer B (0.1% formic acid in 98% acetonitrile, v/v) increased from 6% to 80% at a constant flow rate of 400 nL/min over 40 min (34 min for coelution studies). The eluted peptides were ionized and analyzed by tandem mass spectrometry using the Q-Exactive Plus (Thermo Fisher Scientific) coupled online with ultra-performance liquid chromatography. Full mass spectrometry was performed using the Orbitrap mass analyzer over the range of 350 to 1800 *m/z* with a resolution of 70,000. The 12 most intense ions with a charge ≥2 were fragmented with a normalized collision energy of 28. Tandem mass spectrometry was performed using a mass resolution of 17,500. The first fixed mass was set at 100 *m/z*.

### Proteome and lactylproteome data processing

Raw mass spectrometric data were processed using the MaxQuant environment 66 (version 1.5.2.8) and Andromeda for the database search 67. The tandem mass spectrometry results were matched with the mouse UniProt FASTA database concatenated with the reverse decoy database. Trypsin/P was specified as the cleavage enzyme, allowing for up to four missing cleavages. A label-free proteome analysis was performed using MaxQuant. The mass tolerance for precursor ions was set at 20 ppm during the first search and at 5 ppm during the main search. The mass tolerance for fragment ions was set at 0.02 Da. Carbamidomethyl on Cys was specified as the fixed modification, and lactylation modification and oxidation on Met were specified as variable modifications. For proteome and lactylproteome analyses, when possible, the identity of peptides presents but not sequenced during a given run was obtained by transferring identifications across liquid chromatography mass spectrometry runs. For lactyopeptide identification, an Andromeda minimum score and minimum delta score threshold of 40 and 17 were used, respectively. Up to three missed cleavages were allowed for protease digestion, and the peptide had to be fully tryptic. The false discovery rate was adjusted to < 1%. The minimum score for modified peptides was set at > 40.

### Proteome and lactylproteome bioinformatics data analysis

The bioinformatic analysis was performed using a pipeline written with Perl and R. A statistical analysis of proteome and lactylproteome was performed using logarithmic intensities for those values that were found to be quantified under one experimental condition. To identify significantly modulated proteins and lactyopetides, we performed the three-sample replicate analysis using a *P*-value cutoff of 0.05. Categorical annotations were supplied in the form of Gene Ontology (GO) biological processes (BPs), molecular functions, and cellular components. The Kyoto Encyclopedia of Genes and Genomes (KEGG) enrichment pathway was used for pathway annotation. For each category, a two-tailed Fisher's exact test was used to evaluate the enrichment of the differentially modified protein against all identified proteins. The category with a corrected *P* < 0.05 was considered significant.

### Systemically administration

Given that the drug administration before cerebral ischemia may influence the normal brain metabolism, in order to minimize the impact, we chose to administrate drugs at 90 min before MCAO surgery to allow them were enriched in the brain tissue at the time of cerebral ischemia that can immediately exert inhibiting roles but without much influence to the brain normal brain metabolism. Briefly, 2DG (Sigma, D8375) and sodium oxamate (APExBIO, C3893) was dissolved in sterile saline and administered intraperitoneally at concentrations of 250 mg/kg for 2DG and 15 mg/kg for oxamate in mice at 90 min before MCAO surgery 68. The same volume of sterile saline was used as the vehicle. A-485 (HY-107455; MedChemExpress) was dissolved in corn oil and injected intraperitoneally at a concentration of 50 mg/kg in mice at 90 min before MCAO surgery 48, 69. The vehicle group of mice received the same volume of corn oil. Sodium D-lactate (71716; Sigma Aldrich) or L-lactate (71718; Sigma Aldrich) was dissolved in sterile saline and administered systemically (300 mg/kg) 51, 70 in mice with ischemic stroke immediately after reperfusion.

### Intracerebroventricular injections

Given that the intracerebroventricular injection surgery will damage the brain of mice to some extent, therefore, in order to allow some recovery of mice and meanwhile enrich more lactate to brain tissues before ischemia and without much influence on the normal brain metabolism, we chose to administrate drugs at 24 h before MCAO surgery. Animals were placed in a stereotaxic frame and anesthetized with 2% isoflurane (oxygen flow rate, 2 L/min). Sodium D-lactate (71716; Sigma Aldrich) or L-lactate (71718; Sigma Aldrich) was dissolved in sterile saline and administered (2 μL; 100 mmol/L) 53 intracerebroventricularly (0.46 mm anteroposterior, 1.0 mm lateral, and -2.5 mm dorsoventral to the bregma) in mice at 24 h before MCAO surgery using 26s-gauge needles (Hamilton, Reno, NV, USA). Then, 2 μL of sterile saline was used as the vehicle. In addition, a dose-specific MCT2 inhibitor (α-cyano-4-hydroxycinnamate [4-CIN]; 36 mM in 10 μL of 40% DMSO and 0.9% saline; pH 7.2; HY-107641; MedChemExpress) was injected intracerbroventricularly 39. As a control, 3.6 μL of 40% DMSO was injected. After injection, needles were kept in place for another 5 min and mice were maintained in a 37°C environment until recovery.

### RNA-seq

Brain samples obtained from sham mice and from mice at 6 h after ischemia with preconditioning with vehicle (ischemia 6 h + vehicle) or preconditioning with D-lactate (ischemia 6 h + lactate) were prepared for total RNA extraction and cDNA libraries using the protocol provided by Oxford Nanopore Technologies (ONT) 71. The Super-Script IV First-Strand Synthesis System (Invitrogen, Waltham, MA, USA) was used for full-length mRNA reverse transcription and cDNA PCR amplification for 14 circles using LongAmp Tag. The PCR products were then subjected to FFPE DNA repair and end-repair steps and adaptor ligation using T4 DNA ligase. Agencourt XP beads were used for DNA purification according to the ONT protocol. The final cDNA libraries were added to FLO-MIN109 flow cells and run on the PromethION platform at Biomarker Technology Company (Beijing, China).

Raw reads were first filtered with a minimum average read quality score of 7 and a minimum read length of 500 bp. Ribosomal RNA was discarded after mapping to the rRNA database. Next, full-length, non-chimeric (FLNC) transcripts were determined by searching for primers at both ends of the reads. Clusters of FLNC transcripts were obtained after mapping to the reference genome using mimimap2, and consensus isoforms were obtained after polishing within each cluster using pinfish. Transcripts were validated against known reference transcript annotations using the gffcompare program. A differential expression analysis of two groups was performed using the DESeq R package (1.18.0). DESeq provides statistical routines for determining differential expression in digital gene expression data using a model based on the negative binomial distribution. The resulting *P* values were adjusted using Benjamini and Hochberg's approach for controlling the false discovery rate. Genes with *P* < 0.05 and a fold change ≥1.5 found by DESeq were considered differentially expressed. The GO enrichment analysis of the differentially expressed genes (DEGs) was implemented using the GOseq R package based on Wallenius non-central hypergeometric distribution, which can adjust for gene length bias in DEGs. The statistical enrichment of DEGs in KEGG pathways was performed using KOBAS software 72.

### Immunofluorescent staining

Immunofluorescence (IF) was performed according to the previous report 73. At 6 h after cerebral ischemia or sham surgery, the mice were anesthetized and transcardially perfused with phosphate-buffered saline (PBS) and 4% paraformaldehyde (PFA) to fix their brains. Then, the brains were post-fixed for 24 h in 4% PFA at 4 °C. After cryoprotection using a 15 and 30% sucrose gradient, 10-μm-thick coronal sections were cut using a cryostat microtome (CM1950, Leica). The slices were washed with PBS three times for 5 min and incubated with blocking buffer (0.3% Triton-X-100, 3% BSA in PBS) for 1.5 h at 37 °C, followed by incubation with primary antibodies: goat anti-GFAP antibody (1:200 dilution; ab53554; Abcam), rabbit anti-lactate dehydrogenase antibody (1:200; ab52488; Abcam), mouse anti-NeuN antibody (1:200 dilution; ab104224; Abcam), pan anti-lactyllysine (Kla) antibody (1:500 dilution; PTM-1401; PTM Bio), pan anti-lactyllysine (Kla) antibody (1:500 dilution; PTM-1401RM; PTM Bio), rabbit anti-C3 antibody (1:50 dilution; DF13224; Affbiotech), and rabbit anti-ALDH1L1 antibody (1:200 dilution; Ab177463; Abcam) overnight at 4 °C. After washing three times with PBS, the slices were incubated with fluorescent secondary antibodies (1:200 dilution; SA00003-3, SA00003-1, and SA00009-2; Proteintech) for 1.5 h at 37 °C. Three different brain regions of cortex from three brain sections per animal were analyzed, and the CA1, CA3 and DG region of hippocampus from three brain sections per animal were analyzed in our study. The images were captured using Leica TSC SP8 confocal microscope. Then, the colocalization analysis was performed by ImageJ software.

### qPCR

Total RNA was isolated from cortex with Trizol according to the manufacturer^'^s instruction. One µg of total RNA was reverse transcribed with RT OR-EasyTM II (FOREGENE, FSQ-201). The synthesized cDNA was used as a template for real-time PCR. Semi-quantitative RT-PCR was performed to detected the level of p300 in different groups using β-actin gene as reference. The levels of mRNA were detected by a Bio-Rad cfx maestro system Real-time PCR system (Bio-Rad, USA) by RT-PCR using SYBR®Green Realtime PCR Master Mix (Toyobo, QPK-201). All reactions were performed in triplicates and the relative expression of mRNA was calculated by ^△△^Ct according to standard methods. The primer sequences used for PCR as follows:

p300, forward: 5'-AGCAATGAGTCCCCAAGCTC-3', reverse: 5'-TGTTGCATCATCTGCCGTCT-3'; β-actin, forward: 5'-ATCGTGCGTGACATCAAAGA-3', reverse: 5'-CAAGAAGGAAGGCTGGAAAA-3'.

### Behavioral analysis

According to previously reported methods 74, the adhesive removal test was performed to assess sensorimotor asymmetries. Pieces of 3-mm x 3-mm adhesive tape were applied on the left and right forepaws. Sensorimotor asymmetries were measured by recording the time necessary to remove the adhesive tape from impaired forepaws using the mouth; the maximum observation period was 120 s. Between experiments, animals were housed in home cages in a pathogen-free facility. The test began 1 day before surgery and was repeated at 3, 5, and 7 days after surgery.

According to previously reported methods 75, neurological deficits were evaluated on days 1, 3, 5, and 7 after surgery (0 = no deficit; 1 = forelimb weakness; 2 = circling to the affected side; 3 = partial paralysis on the affected side; and 4 = no spontaneous motor activity). In addition, the body weights of mice were recorded before the evaluation.

### Statistics and reproducibility

The significance of differences in the experimental data was determined using GraphPad Prism 8.0 software. All data involving statistics are presented as the mean ± SEM. Student's t-test was applied for comparisons between two groups, one-way ANOVA for comparisons among multiple groups, and two-way ANOVA with repeated measures were performed when appropriate to compare repeated measured data, and the main effects of the treatment and time points and the interaction were assessed between the two. *P* < 0.05 was considered statistically significant. Two-way ANOVA with repeated measures

### Data availability

The mass spectrometry proteomics data have been deposited at the ProteomeXchange Consortium via the PRIDE 76 partner repository with the dataset identifier PXD019323. The accession number for the RNA-seq data reported in this work is GEO: GSE183439.

## Supplementary Material

Supplementary figures.

Supplementary table 1.

Supplementary table 2.

Supplementary table 3.

Supplementary table 4.

## Abbreviations

2DG: 2-Deoxy-D-glucose
4-CIN: α-cyano-4-hydroxycinnamate
BP: biological process
DEGs: differential expression genes
GO: Gene Ontology
HDAC: histone deacetylases
H&E: hematoxylin and eosin
KEGG: the Kyoto Encyclopedia of Genes and Genomes
Kla: lysine lactylation
LDHA: lactate dehydrogenase A
MCAO: middle cerebral artery occlusion
MCT2: monocarboxylate transporter 2
OXPHO: oxidative phosphorylation
PFA: paraformaldehyde
PTMs: post-translational modifications
TCA: tricarboxylic acid
TTC: 2,3,5-triphenyltetrazolium chloride

## References

[1] Magistretti PJ, Allaman I (2018). Lactate in the brain: from metabolic end-product to signalling molecule. Nat Rev Neurosci.

[2] Allaman I, Magistretti P, Squire L, Berg D, Bloom F, du Lac S (2013). Fundamental Neuroscience. Elsevier Amsterdam, The Netherlands.

[3] Vrselja Z, Daniele SG, Silbereis J, Talpo F, Morozov YM, Sousa AMM (2019). Restoration of brain circulation and cellular functions hours post-mortem. Nature.

[4] Michenfelder JD, Sundt TM Jr (1971). Cerebral ATP and lactate levels in the squirrel monkey following occlusion of the middle cerebral artery. Stroke.

[5] Wang J, Cui Y, Yu Z, Wang W, Cheng X, Ji W (2019). Brain Endothelial Cells Maintain Lactate Homeostasis and Control Adult Hippocampal Neurogenesis. Cell Stem Cell.

[6] Hui S, Ghergurovich JM, Morscher RJ, Jang C, Teng X, Lu W (2017). Glucose feeds the TCA cycle via circulating lactate. Nature.

[7] Castillo X, Rosafio K, Wyss MT, Drandarov K, Buck A, Pellerin L (2015). A probable dual mode of action for both L- and D-lactate neuroprotection in cerebral ischemia. J Cereb Blood Flow Metab.

[8] Walsh CT, Garneau-Tsodikova S, Gatto GJ Jr (2005). Protein posttranslational modifications: the chemistry of proteome diversifications. Angew Chem Int Ed Engl.

[9] Witze ES, Old WM, Resing KA, Ahn NG (2007). Mapping protein post-translational modifications with mass spectrometry. Nat Methods.

[10] Zhao S, Xu W, Jiang W, Yu W, Lin Y, Zhang T (2010). Regulation of cellular metabolism by protein lysine acetylation. Science.

[11] Zhang Z, Tan M, Xie Z, Dai L, Chen Y, Zhao Y (2011). Identification of lysine succinylation as a new post-translational modification. Nat Chem Biol.

[12] Zhang D, Tang Z, Huang H, Zhou G, Cui C, Weng Y (2019). Metabolic regulation of gene expression by histone lactylation. Nature.

[13] Gaffney DO, Jennings EQ, Anderson CC, Marentette JO, Shi T, Schou Oxvig AM (2020). Non-enzymatic Lysine Lactoylation of Glycolytic Enzymes. Cell Chem Biol.

[14] Cui H, Xie N, Banerjee S, Ge J, Jiang D, Dey T (2021). Lung Myofibroblasts Promote Macrophage Profibrotic Activity through Lactate-induced Histone Lactylation. Am J Respir Cell Mol Biol.

[15] Yao X, Li C (2023). Lactate dehydrogenase A mediated histone lactylation induced the pyroptosis through targeting HMGB1. Metabo Brain Dis.

[16] Yao Y, Bade R, Li GT, Zhang AQ, Zhao HL, Fan LF (2023). Global-Scale Profiling of Differential Expressed Lysine-Lactylated Proteins in the Cerebral Endothelium of Cerebral Ischemia-Reperfusion Injury Rats. Cell Mol Neurobiol.

[17] Zhang W, Xu L, Yu ZF, Zhang MQ, Liu JQ, Zhou JM (2023). Inhibition of the Glycolysis Prevents the Cerebral Infarction Progression Through Decreasing the Lactylation Levels of LCP1. Mol Biotechnol.

[18] Combs DJ, Dempsey RJ, Maley M, Donaldson D, Smith C (1990). Relationship between plasma glucose, brain lactate, and intracellular pH during cerebral ischemia in gerbils. Stroke.

[19] Frerichs KU, Lindsberg PJ, Hallenbeck JM, Feuerstein GZ (1990). Increased cerebral lactate output to cerebral venous blood after forebrain ischemia in rats. Stroke.

[20] Cvoro V, Wardlaw JM, Marshall I, Armitage PA, Rivers CS, Bastin ME (2009). Associations between diffusion and perfusion parameters, N-acetyl aspartate, and lactate in acute ischemic stroke. Stroke.

[21] Graham GD, Blamire AM, Howseman AM, Rothman DL, Fayad PB, Brass LM (1992). Proton magnetic resonance spectroscopy of cerebral lactate and other metabolites in stroke patients. Stroke.

[22] Munoz Maniega S, Cvoro V, Chappell FM, Armitage PA, Marshall I, Bastin ME (2008). Changes in NAA and lactate following ischemic stroke: a serial MR spectroscopic imaging study. Neurology.

[23] Wu X, Zhao H, Min L, Zhang C, Liu P, Luo Y (2014). Effects of 2-Deoxyglucose on ischemic brain injuries in rats. Int J Neurosci.

[24] Bosoi CR, Zwingmann C, Marin H, Parent-Robitaille C, Huynh J, Tremblay M (2014). Increased brain lactate is central to the development of brain edema in rats with chronic liver disease. J Hepatol.

[25] Arumugam TV, Baik SH, Balaganapathy P, Sobey CG, Mattson MP, Jo DG (2018). Notch signaling and neuronal death in stroke. Prog Neurobiol.

[26] Liddelow SA, Guttenplan KA, Clarke LE, Bennett FC, Bohlen CJ, Schirmer L (2017). Neurotoxic reactive astrocytes are induced by activated microglia. Nature.

[27] Lan X, Han X, Li Q, Yang QW, Wang J (2017). Modulators of microglial activation and polarization after intracerebral haemorrhage. Nat Rev Neurol.

[28] Zhang Y, Chen K, Sloan SA, Bennett ML, Scholze AR, O'Keeffe S (2014). An RNA-sequencing transcriptome and splicing database of glia, neurons, and vascular cells of the cerebral cortex. J Neurosci.

[29] Liang YJ, Yang YR, Tao CY, Yang SH, Zhang XX, Yuan J (2022). Deep Succinylproteomics of Brain Tissues from Intracerebral Hemorrhage with Inhibition of Toll-Like Receptor 4 Signaling. Cell Mol Neurobiol.

[30] Ravindran E, Arashiki N, Becker LL, Takizawa K, Lévy J, Rambaud T (2022). Monoallelic CRMP1 gene variants cause neurodevelopmental disorder. Elife.

[31] Higurashi M, Iketani M, Takei K, Yamashita N, Aoki R, Kawahara N (2012). Localized role of CRMP1 and CRMP2 in neurite outgrowth and growth cone steering. Dev Neurobiol.

[32] Kawashima T, Jitsuki-Takahashi A, Takizawa K, Jitsuki S, Takahashi T, Ohshima T (2021). Phosphorylation of Collapsin Response Mediator Protein 1 (CRMP1) at Tyrosine 504 residue regulates Semaphorin 3A-induced cortical dendritic growth. J Neurochem.

[33] Schmidt EF, Strittmatter SM (2007). The CRMP family of proteins and their role in Sema3A signaling. Adv Exp Med Biol.

[34] Li WX, Cheng TL, Dong XR, Chen HY, Yang L, Qiu ZL (2022). KIF5C deficiency causes abnormal cortical neuronal migration, dendritic branching, and spine morphology in mice. Pediatr Res.

[35] Corsi A, Bombieri C, Valenti MT, Romanelli MG (2022). Tau Isoforms: Gaining Insight into Alternative Splicing. Int J Mol Sci.

[36] Simone R, Javad F, Emmett W, Wilkins OG, Almeida FL, Barahona-Torres N (2021). MIR-NATs repress translation and aid proteostasis in neurodegeneration. Nature.

[37] Yi S, Liu QY, Wang XH, Qian TM, Wang HK, Zha GB (2019). Tau modulates Schwann cell proliferation, migration and differentiation following peripheral nerve injury. J Cell Sci.

[38] Newman LA, Korol DL, Gold PE (2011). Lactate produced by glycogenolysis in astrocytes regulates memory processing. PLoS One.

[39] Matsui T, Omuro H, Liu YF, Soya M, Shima T, McEwen BS (2017). Astrocytic glycogen-derived lactate fuels the brain during exhaustive exercise to maintain endurance capacity. Proc Natl Acad Sci U S A.

[40] Dienel GA, Cruz NF (2016). Aerobic glycolysis during brain activation: adrenergic regulation and influence of norepinephrine on astrocytic metabolism. J Neurochem.

[41] Goyal MS, Hawrylycz M, Miller JA, Snyder AZ, Raichle ME (2014). Aerobic glycolysis in the human brain is associated with development and neotenous gene expression. Cell Metab.

[42] Pierre K, Pellerin L (2005). Monocarboxylate transporters in the central nervous system: distribution, regulation and function. J Neurochem.

[43] Moreno-Yruela C, Zhang D, Wei W, Bæk M, Liu WC, Gao JJ (2022). Class I histone deacetylases (HDAC1-3) are histone lysine delactylases. Sci Adv.

[44] Liao YJ, Cheng JB, Kong XX, Li SS, Li XH, Zhang MJ (2020). HDAC3 inhibition ameliorates ischemia/reperfusion-induced brain injury by regulating the microglial cGAS-STING pathway. Theranostics.

[45] Lu H, Ashiqueali R, Lin CI, Walchale A, Clendaniel V, Matheson R (2023). Histone Deacetylase 3 Inhibition Decreases Cerebral Edema and Protects the Blood-Brain Barrier After Stroke. Mol Neurobiol.

[46] Zhang MJ, Zhao QC, Xia MX, Chen J, Chen YT, Cao X (2020). The HDAC3 inhibitor RGFP966 ameliorated ischemic brain damage by downregulating the AIM2 inflammasome. FASEB J.

[47] Yang XY, Wu QM, Zhang L, Feng LY (2016). Inhibition of Histone Deacetylase 3 (HDAC3) Mediates Ischemic Preconditioning and Protects Cortical Neurons against Ischemia in Rats. Front Mol Neurosci.

[48] Peng J, Li J, Huang J, Xu P, Huang H, Liu Y (2019). p300/CBP inhibitor A-485 alleviates acute liver injury by regulating macrophage activation and polarization. Theranostics.

[49] Lasko LM, Jakob CG, Edalji RP, Qiu W, Montgomery D, Digiammarino EL (2017). Discovery of a selective catalytic p300/CBP inhibitor that targets lineage-specific tumours. Nature.

[50] Li HH, Guglielmetti C, Sei YJ, Zilberter M, Le Page LM, Shields L (2023). Neurons require glucose uptake and glycolysis in vivo. Cell Rep.

[51] Lev-Vachnish Y, Cadury S, Rotter-Maskowitz A, Feldman N, Roichman A, Illouz T (2019). L-Lactate Promotes Adult Hippocampal Neurogenesis. Front Neurosci.

[52] Sada N, Lee S, Katsu T, Otsuki T, Inoue T (2015). Epilepsy treatment. Targeting LDH enzymes with a stiripentol analog to treat epilepsy. Science.

[53] Berthet C, Lei H, Thevenet J, Gruetter R, Magistretti PJ, Hirt L (2009). Neuroprotective role of lactate after cerebral ischemia. J Cereb Blood Flow Metab.

[54] Berthet C, Castillo X, Magistretti PJ, Hirt L (2012). New evidence of neuroprotection by lactate after transient focal cerebral ischaemia: extended benefit after intracerebroventricular injection and efficacy of intravenous administration. Cerebrovasc Dis.

[55] Irizarry-Caro RA, McDaniel MM, Overcast GR, Jain VG, Troutman TD, Pasare C (2020). TLR signaling adapter BCAP regulates inflammatory to reparatory macrophage transition by promoting histone lactylation. Proc Natl Acad Sci U S A.

[56] Jiang J, Huang D, Jiang Y, Hou J, Tian M, Li J (2021). Lactate Modulates Cellular Metabolism Through Histone Lactylation-Mediated Gene Expression in Non-Small Cell Lung Cancer. Front Oncol.

[57] Yu J, Chai P, Xie M, Ge S, Ruan J, Fan X (2021). Histone lactylation drives oncogenesis by facilitating m(6)A reader protein YTHDF2 expression in ocular melanoma. Genome Biol.

[58] Gao M, Zhang N, Liang W (2020). Systematic Analysis of Lysine Lactylation in the Plant Fungal Pathogen Botrytis cinerea. Front Microbiol.

[59] An W, Kim J, Roeder RG (2004). Ordered cooperative functions of PRMT1, p300, and CARM1 in transcriptional activation by p53. Cell.

[60] Wang ZA, Cole PA (2020). The Chemical Biology of Reversible Lysine Post-translational Modifications. Cell Chem Biol.

[61] Wang YH, Israelsen WJ, Lee D, Yu VWC, Jeanson NT, Clish CB (2014). Cell-state-specific metabolic dependency in hematopoiesis and leukemogenesis. Cell.

[62] Srinivasan R, Lu TY, Chai H, Xu J, Huang BS, Golshani P (2016). New Transgenic Mouse Lines for Selectively Targeting Astrocytes and Studying Calcium Signals in Astrocyte Processes In Situ and In Vivo. Neuron.

[63] Wang PF, Fang H, Chen J, Lin S, Liu Y, Xiong XY (2014). Polyinosinic-polycytidylic acid has therapeutic effects against cerebral ischemia/reperfusion injury through the downregulation of TLR4 signaling via TLR3. J Immunol.

[64] Xiong XY, Liu L, Wang FX, Yang YR, Hao JW, Wang PF (2016). Toll-Like Receptor 4/MyD88-Mediated Signaling of Hepcidin Expression Causing Brain Iron Accumulation, Oxidative Injury, and Cognitive Impairment After Intracerebral Hemorrhage. Circulation.

[65] Yuan JJ, Zhang Q, Gong CX, Wang FX, Huang JC, Yang GQ (2019). Young plasma ameliorates aging-related acute brain injury after intracerebral hemorrhage. Biosci Rep.

[66] Cox J, Mann M (2008). MaxQuant enables high peptide identification rates, individualized p.p.b.-range mass accuracies and proteome-wide protein quantification. Nat Biotechnol.

[67] Cox J, Neuhauser N, Michalski A, Scheltema RA, Olsen JV, Mann M (2011). Andromeda: a peptide search engine integrated into the MaxQuant environment. J Proteome Res.

[68] Garcia-Castillo V, Lopez-Urrutia E, Villanueva-Sanchez O, Avila-Rodriguez MA, Zentella-Dehesa A, Cortes-Gonzalez C (2017). Targeting Metabolic Remodeling in Triple Negative Breast Cancer in a Murine Model. J Cancer.

[69] Zhou F, Liu Q, Zhang L, Zhu Q, Wang S, Zhu K (2020). Selective inhibition of CBP/p300 HAT by A-485 results in suppression of lipogenesis and hepatic gluconeogenesis. Cell Death Dis.

[70] Won SJ, Jang BG, Yoo BH, Sohn M, Lee MW, Choi BY (2012). Prevention of acute/severe hypoglycemia-induced neuron death by lactate administration. J Cereb Blood Flow Metab.

[71] Lu H, Giordano F, Ning Z (2016). Oxford Nanopore MinION Sequencing and Genome Assembly. Genomics Proteomics Bioinformatics.

[72] Bu D, Luo H, Huo P, Wang Z, Zhang S, He Z (2021). KOBAS-i: intelligent prioritization and exploratory visualization of biological functions for gene enrichment analysis. Nucleic Acids Res.

[73] Hagihara H, Shoji H, Otabi H, Toyoda A, Katoh K, Namihira M (2021). Protein lactylation induced by neural excitation. Cell Rep.

[74] Xu J, Chen Z, Yu F, Liu H, Ma C, Xie D (2020). IL-4/STAT6 signaling facilitates innate hematoma resolution and neurological recovery after hemorrhagic stroke in mice. Proc Natl Acad Sci U S A.

[75] Sun J, Huang Y, Gong J, Wang J, Fan Y, Cai J (2020). Transplantation of hPSC-derived pericyte-like cells promotes functional recovery in ischemic stroke mice. Nat Commun.

[76] Perez-Riverol Y, Csordas A, Bai J, Bernal-Llinares M, Hewapathirana S, Kundu DJ (2019). The PRIDE database and related tools and resources in 2019: improving support for quantification data. Nucleic Acids Res.

