# Supplementary material for: Astrocyte-derived lactate aggravates brain injury of ischemic stroke in mice by promoting the formation of protein lactylation
Source: Theranostics. 2024 Jul 8;14(11):4297–317. doi: 10.7150/thno.96375 (PMC11303085; doi:10.7150/thno.96375)
Supplement: Supplementary file 1 — Supplementary figures. [file thnov14p4297s1.pdf]

Supplemental Figures and Figure Legends

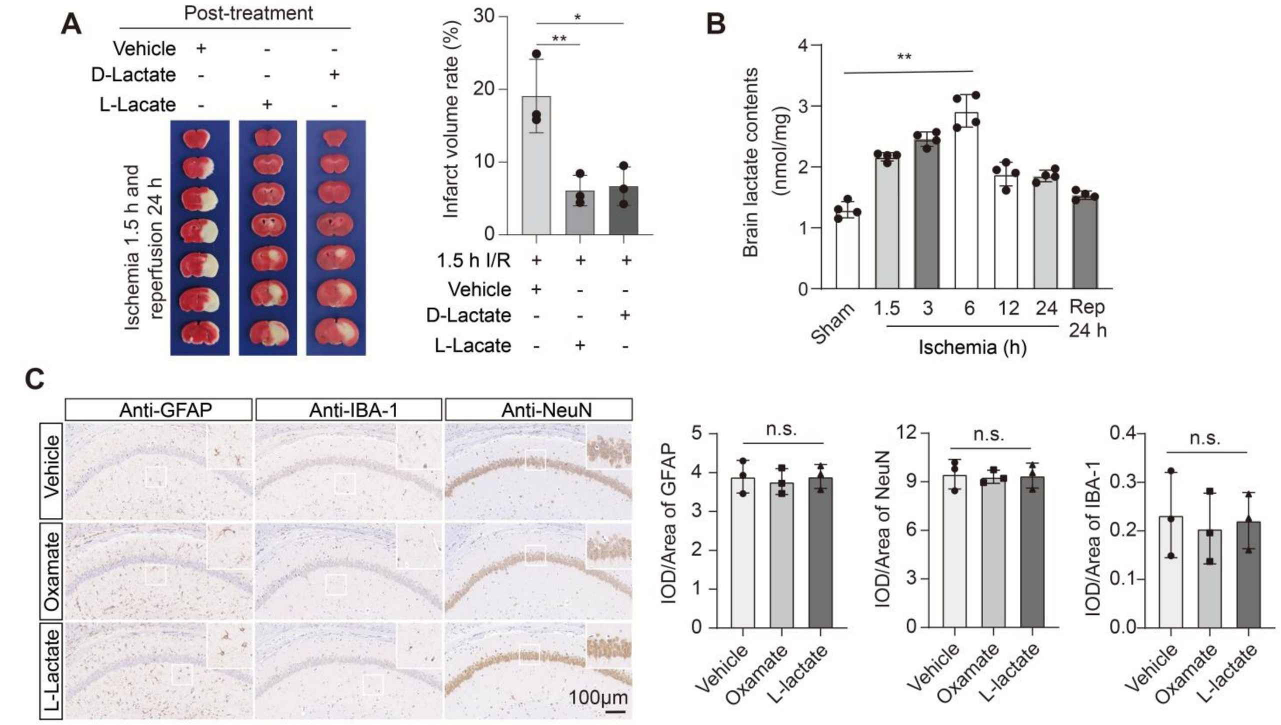

Figure S1. Ischemic stroke and the influence of drugs on normal brains. A, Representative image of 2,3,5-triphenyltetrazolium chloride (TTC) staining of brain sections at 24 h reperfusion after 1.5 h of ischemia with lactate treatment immediately after reperfusion (n = 3). **B,** Ischemic brain levels of lactate at different times of ischemic stroke (n = 4). **C**, Representative images and statistical analysis of immunohistochemical staining show the expression levels of neuroglial markers of NeuN, GFAP, and IBa-1 in the normal hippocampus of mice treated with Oxamate, L-lactate and vehicle, respectively (n = 3). *$P < 0.05$, **$P < 0.01$, and n.s. indicates a non-significant difference.

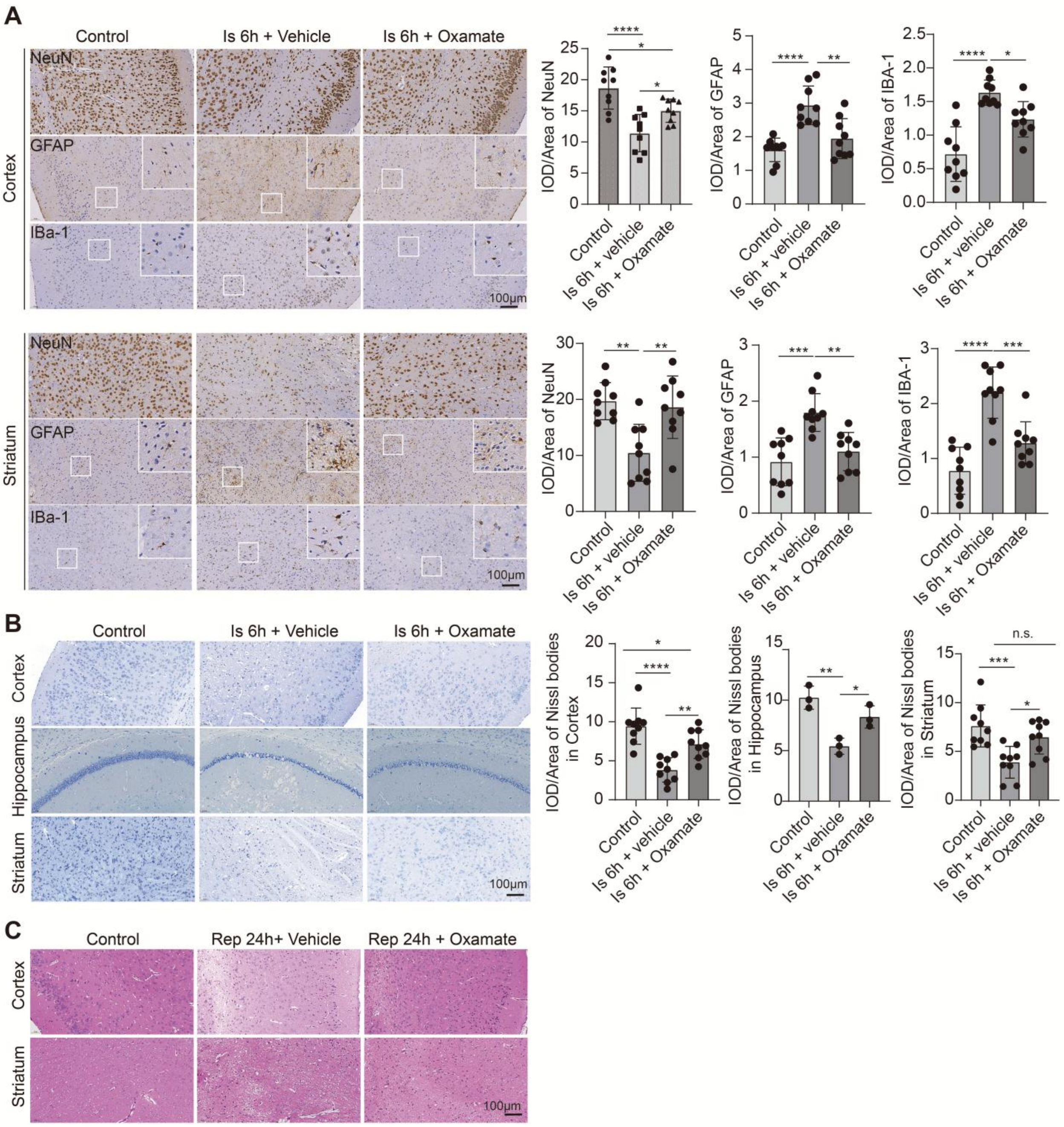

Figure S2. Representative images of brain injury in mice with ischemic stroke and Oxamate preconditioning treatment and lactate post-treatment. A, Representative images and statistical analysis of immunohistochemical staining show the expression levels of neuroglial markers of NeuN, GFAP, and IBa-1 in the ischemic cortex and striatum at 6 h post cerebral ischemia after Oxamate preconditioning (n = 3). **B,** Representative images and statistical analysis of Nissl staining in the ischemic cortex, hippocampus CA1 region, and striatum at 6 h post cerebral ischemia after Oxamate preconditioning (n = 3). **C,** Representative images of hematoxylin and eosin (H&E)

staining show damage in the ischemic hippocampus and striatum at 24 h of reperfusion after 1.5 h of ischemia preconditioning (n = 3). Scale bar = 100 μm. *$P < 0.05$, **$P < 0.01$, ***$P < 0.001$ and ****$P < 0.0001$; n.s. indicates a non-significant difference.

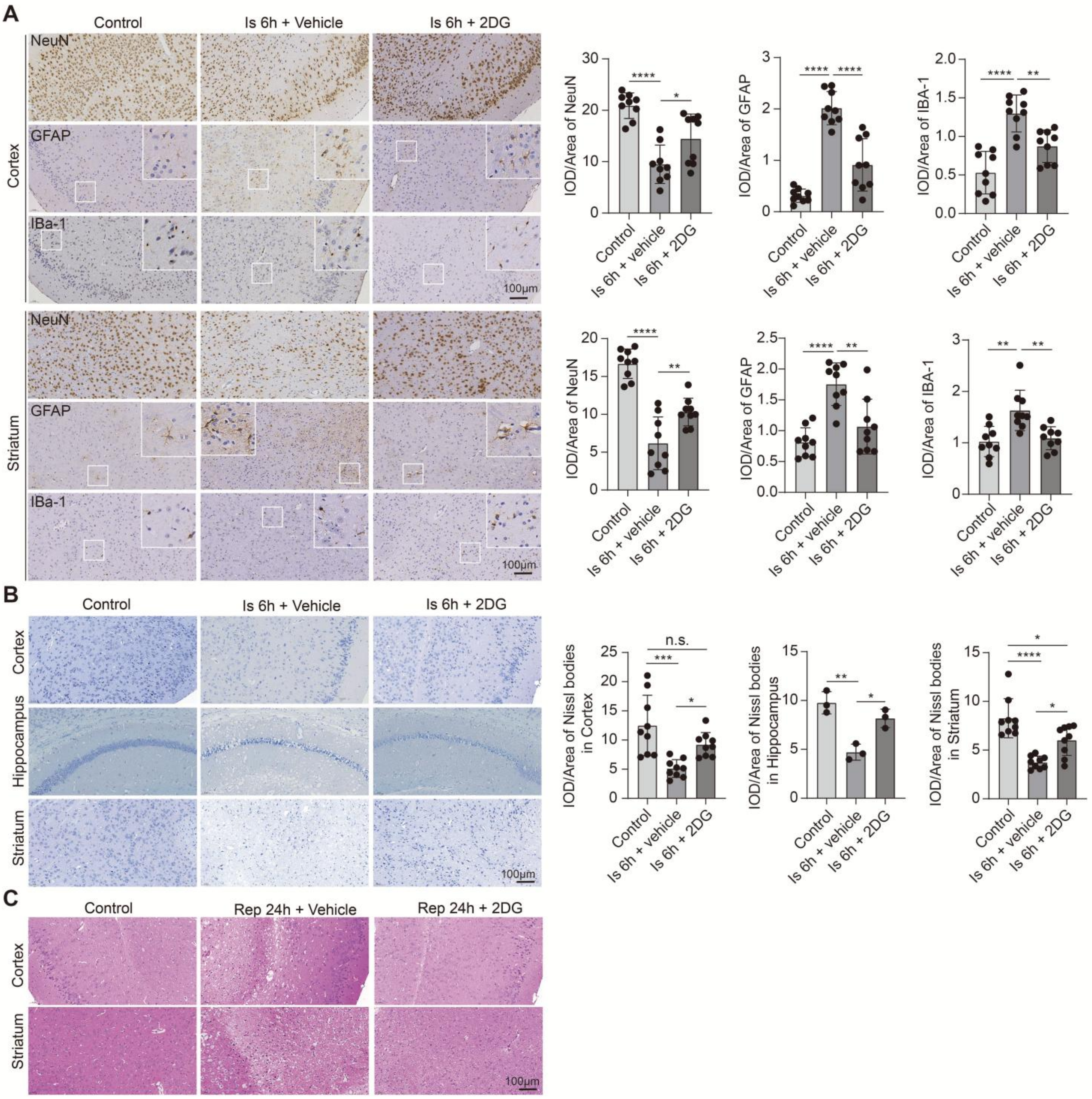

Figure S3. Representative images of brain injury in mice with ischemic stroke preconditioning treated with 2DG. A, Representative images and statistical analysis of immunohistochemical staining show the expression levels of neuroglial markers of NeuN, GFAP, and IBa-1 in the ischemic cortex and striatum at 6 h post cerebral ischemia after 2DG preconditioning (n = 3). **B,** Representative images and statistical analysis of Nissl staining in the ischemic cortex, hippocampus CA1 region, and striatum at 6 h post cerebral ischemia after 2DG preconditioning (n = 3). **C,** Representative images of hematoxylin and eosin (H&E) staining show damage in the cortex and

striatum at 24 h of reperfusion after 1.5 h ischemia (n = 3). Scale bar = 100 μm. $^*P < 0.05$, $^{**}P < 0.01$, $^{***}P < 0.001$ and $^{****}P < 0.0001$.

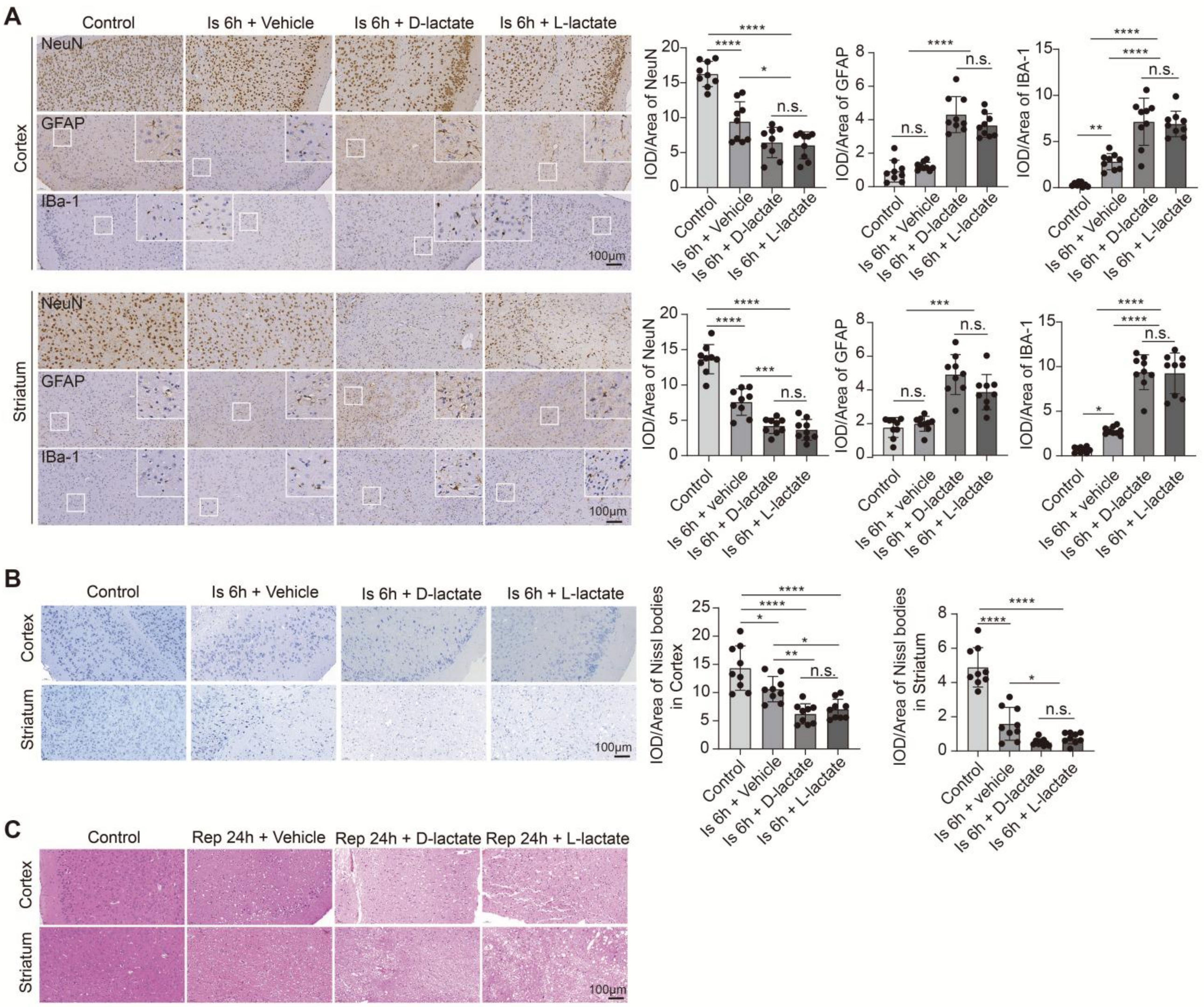

Figure S4. Representative images of brain injury in mice with ischemic stroke and lactate preconditioning treatment. A, Representative images and statistical analysis of immunohistochemical staining show the expression levels of neuroglial markers of NeuN, GFAP, and IBa-1 in the ischemic cortex and striatum after 6 h of cerebral ischemia and lactate preconditioning (n = 3). **B,** Representative images and statistical analysis of Nissl staining in the ischemic cortex and striatum after 6 h of cerebral ischemia and lactate preconditioning (n = 3). **C,** Representative images of hematoxylin and eosin (H&E) staining show damage in the ischemic cortex and striatum at 24 h of reperfusion after 1.5 h of ischemia with lactate preconditioning (n = 3). Scale bar = 100 μm. **P* < 0.05, ***P* < 0.01, ****P* < 0.001 and *****P* < 0.0001; n.s. indicates a non-significant difference.

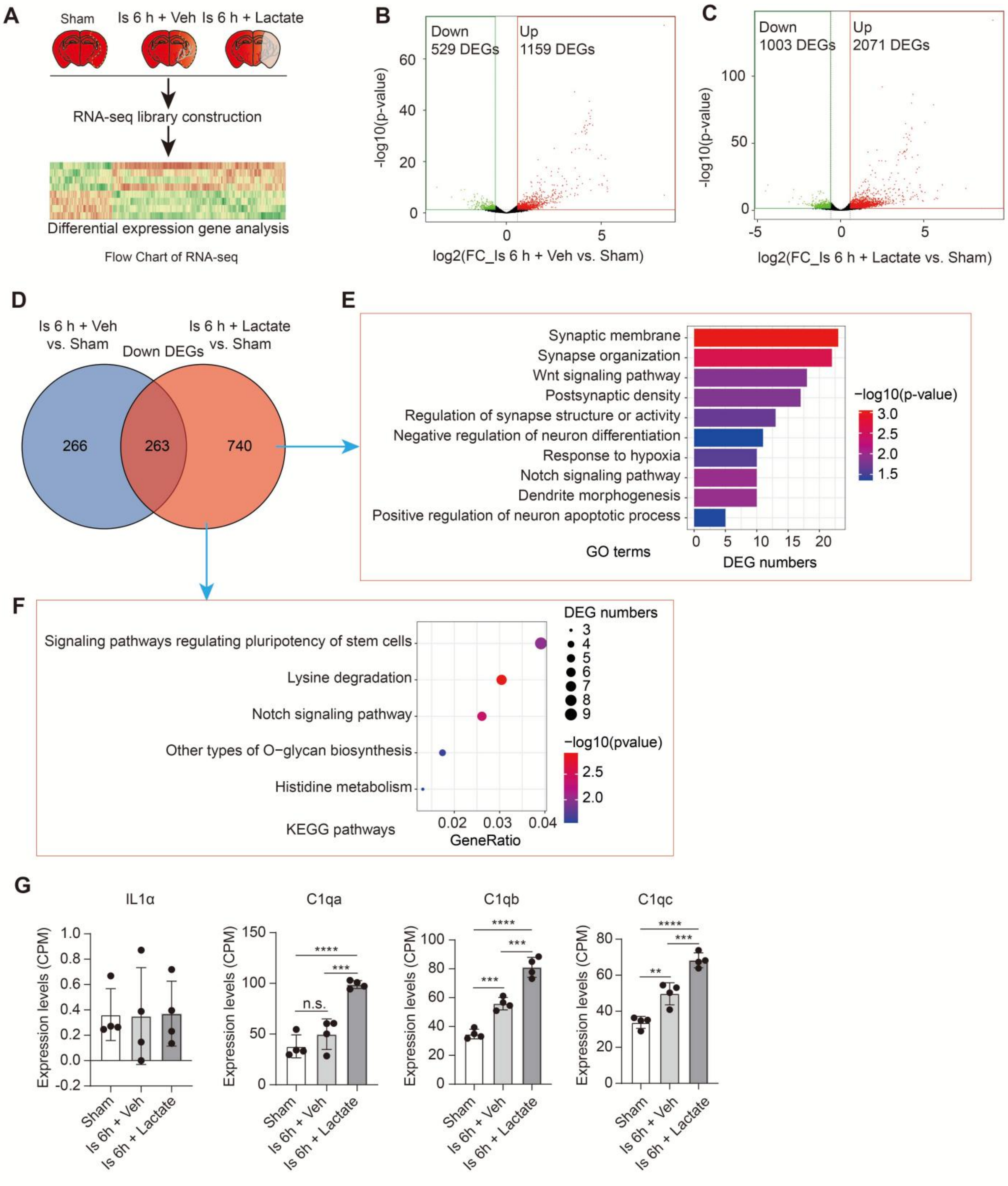

Figure S5. Analysis of transcriptomic data. A, Schematic of the experimental strategy used to identify the differentially expressed genes (DEGs) induced by cerebral ischemia with or without lactate preconditioning treatment. **B-C,** Volcano plot of gene expression changes in ischemic brains from vehicle-treated mice compared with sham mice (B) and from lactate-treated mice compared with sham mice (C). **D,** Venn diagram shows overlapped and specific downregulated DEGs between the lactate-treated and vehicle-

treated ischemic brain tissues at 6 h after cerebral ischemia. **E,** The Gene Ontology (GO) biological process (BP) enrichment pathways of the specific lactate treatment downregulated DEGs. **F,** The Kyoto Encyclopedia of Genes and Genomes (KEGG) enrichment pathways of the specific lactate treatment downregulated DEGs. (n = 4 for each group). **G,** Expression levels of inflammatory cytokines in ischemic brain tissues treated with or without lactate (n = 4). $^{**}P < 0.01$, $^{***}P < 0.001$ and $^{****}P < 0.0001$.

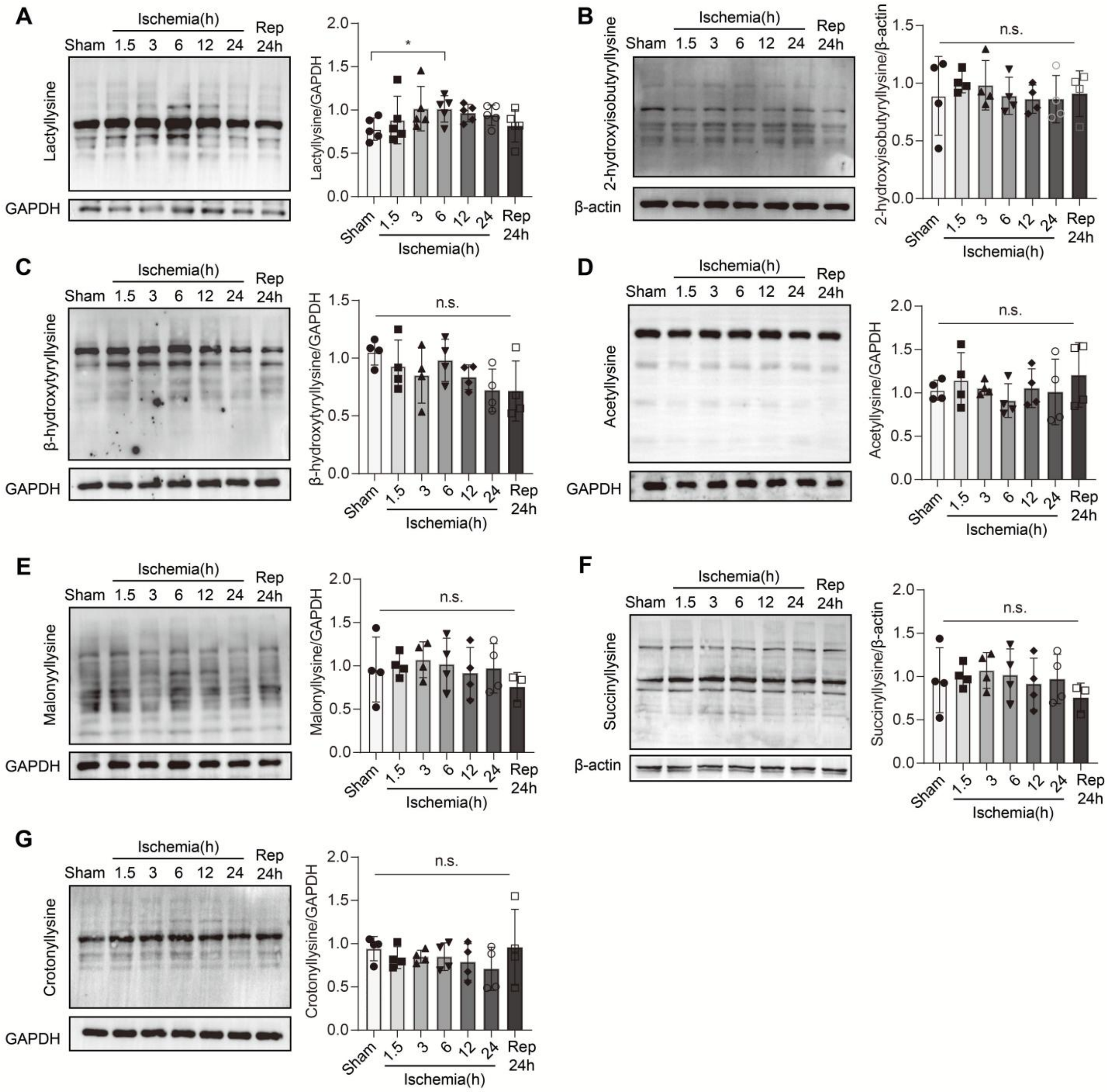

Figure S6. Western blot analysis of protein post-translational modifications (PTMs) of brains after ischemic stroke. A-G, Changes in the expression levels of brain protein lactylation (A), acetylation (B), succinylation (C), crotonylation (D), 2-hydroxyisobutyrylation (E), β-hydroxybutyrylation (F), and β-hydroxybutyrylation (G) in mice with ischemic stroke at different times as detected by the western blot analysis. n = 4 for each group. *$P < 0.05$, n.s. indicates a non-significant difference.

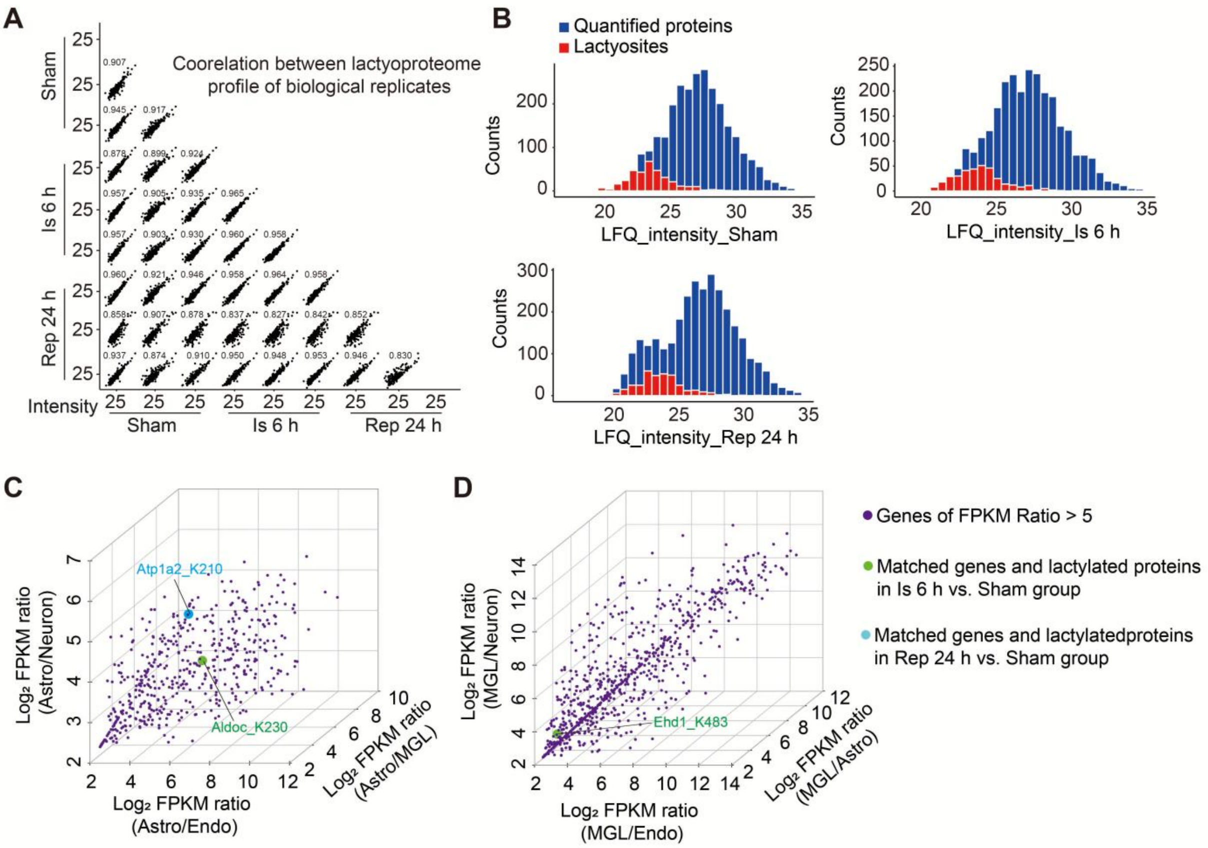

Figure S7. Analysis of lactylproteome data. A, Comparison between biological replicates of mass spectrometry-based lactylproteomics experiments involving the cerebral cortex from the sham, 6-h ischemia, and 24-h reperfusion groups. Each dot represents one protein. The Pearson correlation coefficients are indicated in each scatterplot. **B,** The distribution of ranked label-free quantitation (LFQ) intensity values of the sham, 6-h ischemia, and reperfusion groups is coded using blue and lactylation intensity is coded using red. **C-D,** Three-dimensional scatter plots show some of the significant changes in lactylated proteins to be mainly distributed in astrocytes (C) and microglia (D). Matching the significant changes in lactylated proteins ($P < 0.05$, Student's *t*-test) with the relatively specific higher-expression genes of neural cells (gene expression in one of the four neural cell types was five-times higher than that of other cellular types) from the published transcriptome data (n = 3 for each group).

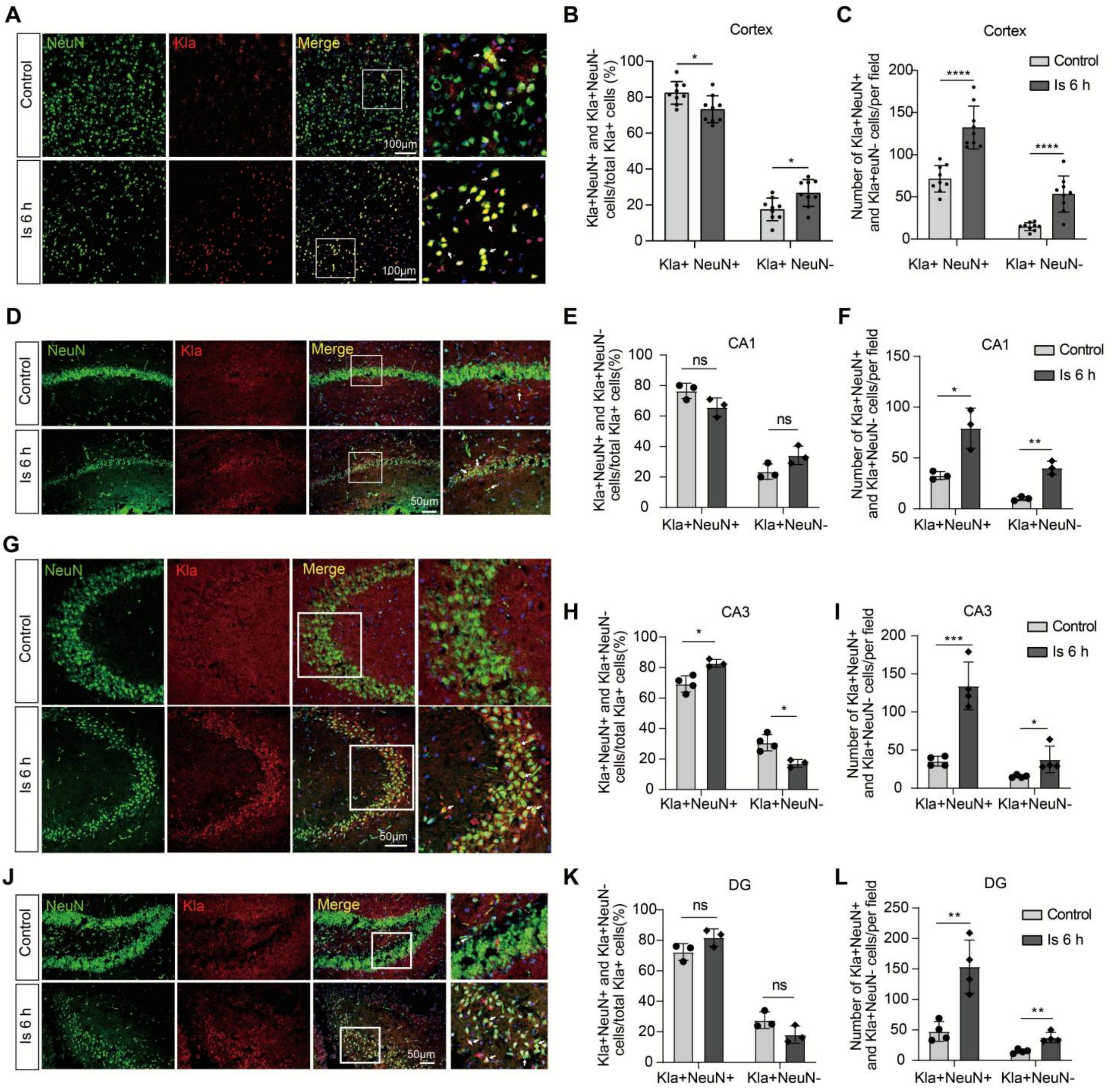

Figure S8. Representative images of increased protein lactylation levels with NeuN+ neurons after 6 h of cerebral ischemia. A, Representative image of colocalization staining with Kla (red) and NeuN+ neurons (green) in ischemic cortex. **B,** Statistic analysis of the percentage of the Kla+NeuN+ and Kla+NeuN- cells/total Kla+ cells in ischemic cortex. **C,** Statistic analysis of the number of Kla+NeuN+ and Kla+NeuN- cells in per field of ischemic cortex. Three locations within the infarct area were randomly selected for representing the ischemic cortex of each brain slice, the corresponding contralateral brain tissues were selected as the control groups. Then, the cellular information of each location were calculated, which is the per field of ischemic

cortex. **D,** Representative image of colocalization staining with Kla (red) and NeuN+ neurons (green) in ischemic CA1 of hippocampus. **E,** Statistic analysis of the percentage of the Kla+NeuN+ and Kla+NeuN- cells/total Kla+ cells in ischemic CA1 of hippocampus. **F,** Statistic analysis of the number of Kla+NeuN+ and Kla+NeuN- cells in ischemic CA1 of hippocampus. **G,** Representative image of colocalization staining with Kla (red) and NeuN+ neurons (green) in ischemic CA3 of hippocampus. **H,** Statistic analysis of the percentage of the Kla+NeuN+ and Kla+NeuN- cells/total Kla+ cells in ischemic CA3 of hippocampus. **I,** Statistic analysis of the number of Kla+NeuN+ and Kla+NeuN- cells in ischemic CA3 of hippocampus. **J,** Representative image of colocalization staining with Kla (red) and NeuN+ neurons (green) in ischemic DG of hippocampus. **K,** Statistic analysis of the percentage of the Kla+NeuN+ and Kla+NeuN- cells/total Kla+ cells in ischemic DG of hippocampus. **L,** Statistic analysis of the number of Kla+NeuN+ and Kla+NeuN- cells in ischemic DG of hippocampus.n = 3 for each group, Scale bar = 100 μm for a, = 50 for d, g,and j. *$P < 0.05$, **$P < 0.01$, ***$P < 0.001$, and ****$P < 0.0001$. n.s. indicates a non-significant difference.

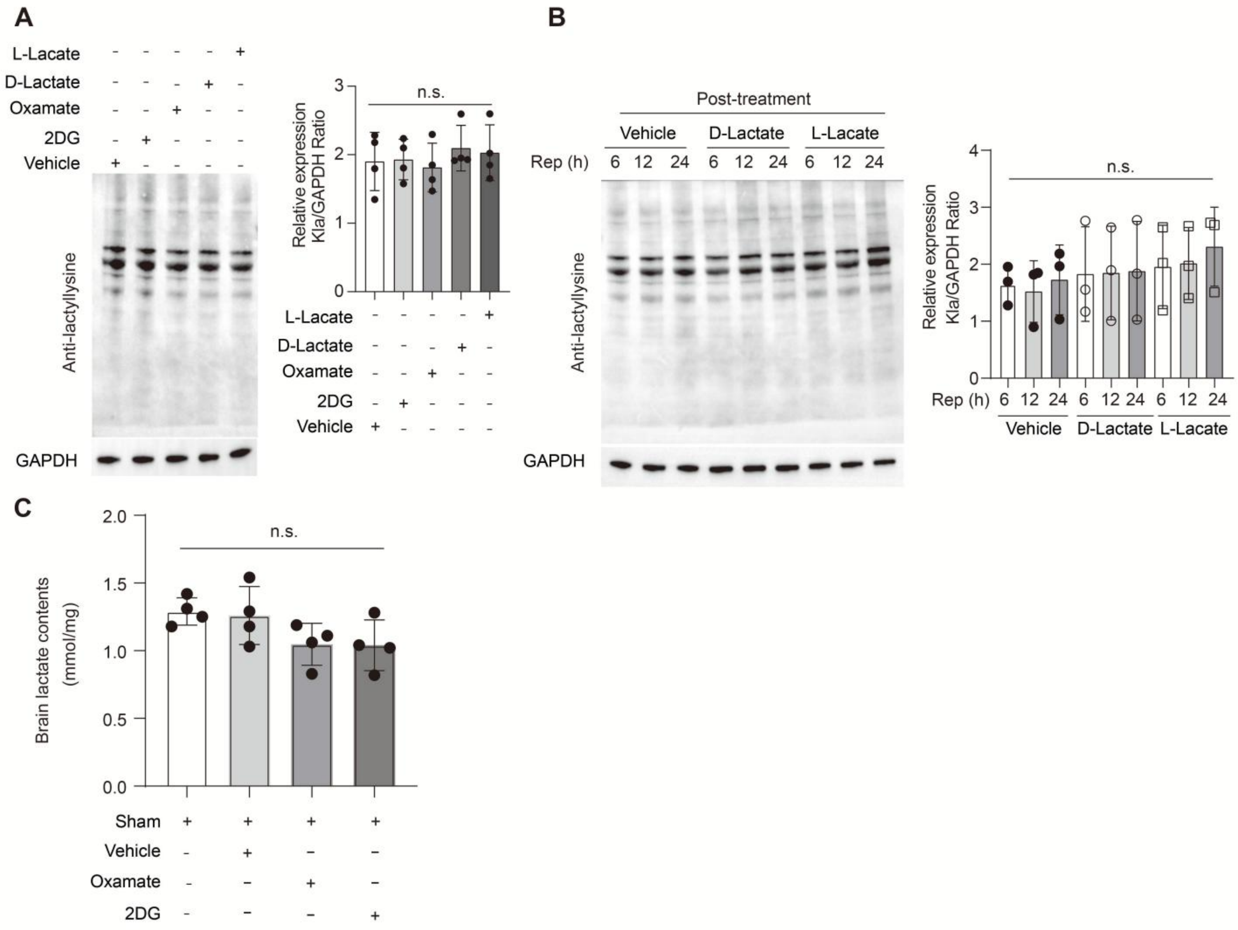

Figure S9. Regulation of brain lactate levels significantly alters protein lactylation levels of ischemic brain tissue. A, Preconditioning D-lactate, L-lactate, 2-deoxyglucose (2DG), and oxamate treatment do not change the protein lactylation levels of brain tissues without cerebral ischemia (n = 4). **B,** Post-treatment with D-lactate and L-lactate via intraperitoneal injection immediately after reperfusion does not change the protein lactylation levels of brain tissues at 6, 12, and 24 h after reperfusion (n = 3). **C,** Lactate levels in the brain of mice at 6 h after sham surgery were measured after preconditioning 2DG and oxamate treatments administered via intraperitoneal injection (n = 4).

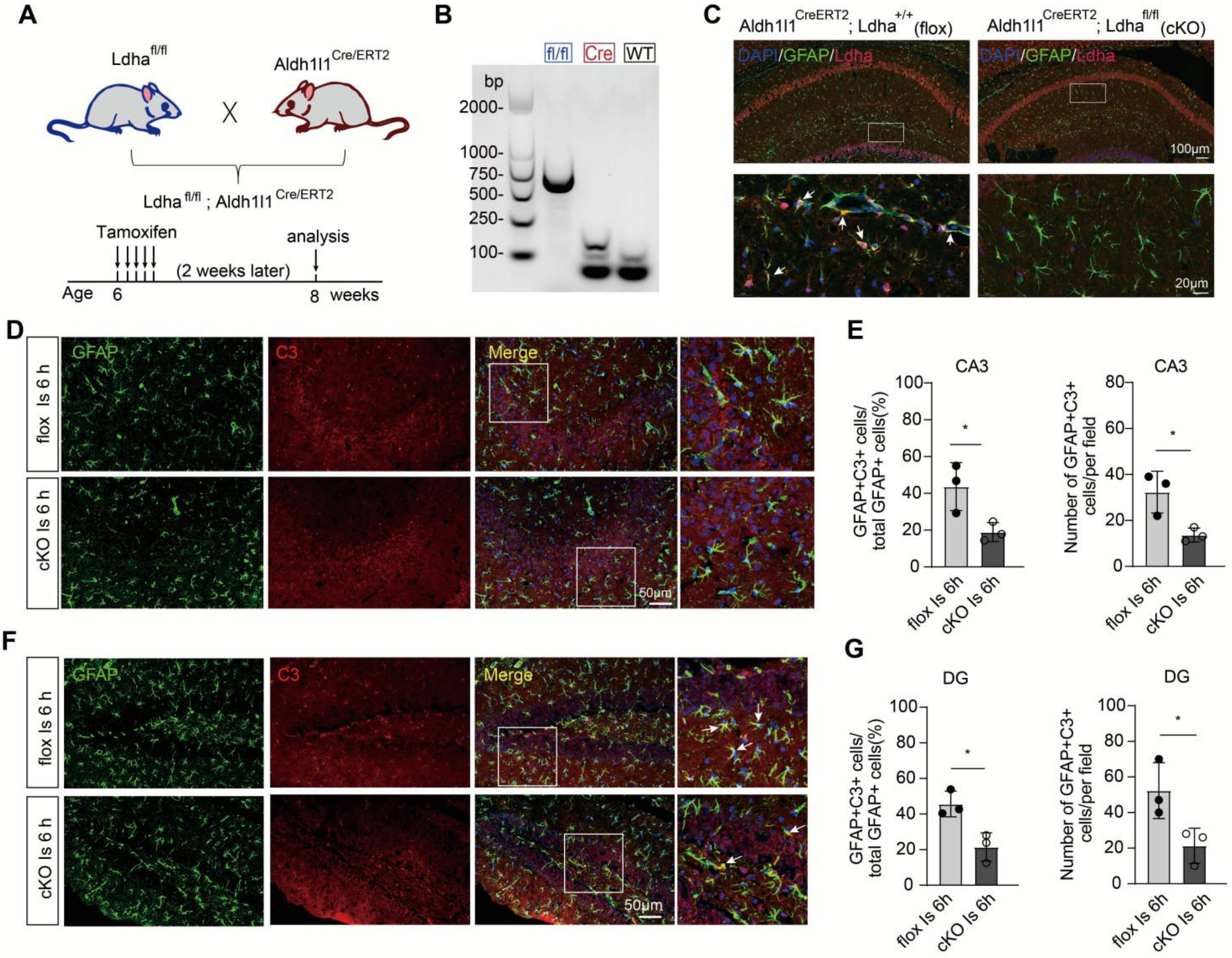

Figure S10. ***Aldh1l1*$^{\text{CreERT2}}$; *Ldha*$^{\text{fl/fl}}$ mice showed better outcomes of ischemic stroke. A,** Schematic of the approach for the construction of *Aldh1l1*$^{\text{CreERT2}}$; *Ldha*$^{\text{fl/fl}}$ mice. **B,** Genotyping of Ldha$^{\text{flox/flox}}$ (Ldha$^{\text{fl/fl}}$) mice and Aldh1l1$^{\text{CreERT2}}$ mice with their corresponding primers according to the manufacturers protocol. Genotyping of wild-type (WT) mice was used as a negative control. **C,** Representative hippocampal images confirm the successful construction of *Aldh1l1*$^{\text{Cre/ERT2}}$; *Ldha*$^{\text{fl/fl}}$ transgenic mouse stained for GFAP (green) and Ldha (red) after induced by tamoxifen (100 µl tamoxifen per mouse for 5 consecutive days). **D,** Representative image of colocalization staining with GFAP+ astrocytes (green) and C3 (red) in CA3 region of hippocampus of ischemic cKO mice compared to ischemic flox mice. **E,** Statistic analysis of the percentage of the GFAP+C3+/GFAP+ cells and number of GFAP+C3+ cells per field in CA3 region of hippocampus of ischemic cKO mice compared to ischemic flox mice. **F,** Representative image of colocalization staining with GFAP+ astrocytes (green) and C3

(red) in DG region of hippocampus of ischemic cKO mice compared to ischemic flox mice. **G,** Statistic analysis of the percentage of the GFAP+C3+/GFAP+ cells and number of GFAP+C3+ cells per field in DG region of hippocampus of ischemic cKO mice compared to ischemic flox mice. n = 3 for each group. Scale bar = 100 μm and 20 μm for C, = 50 μm for d and f. *$P < 0.05$.

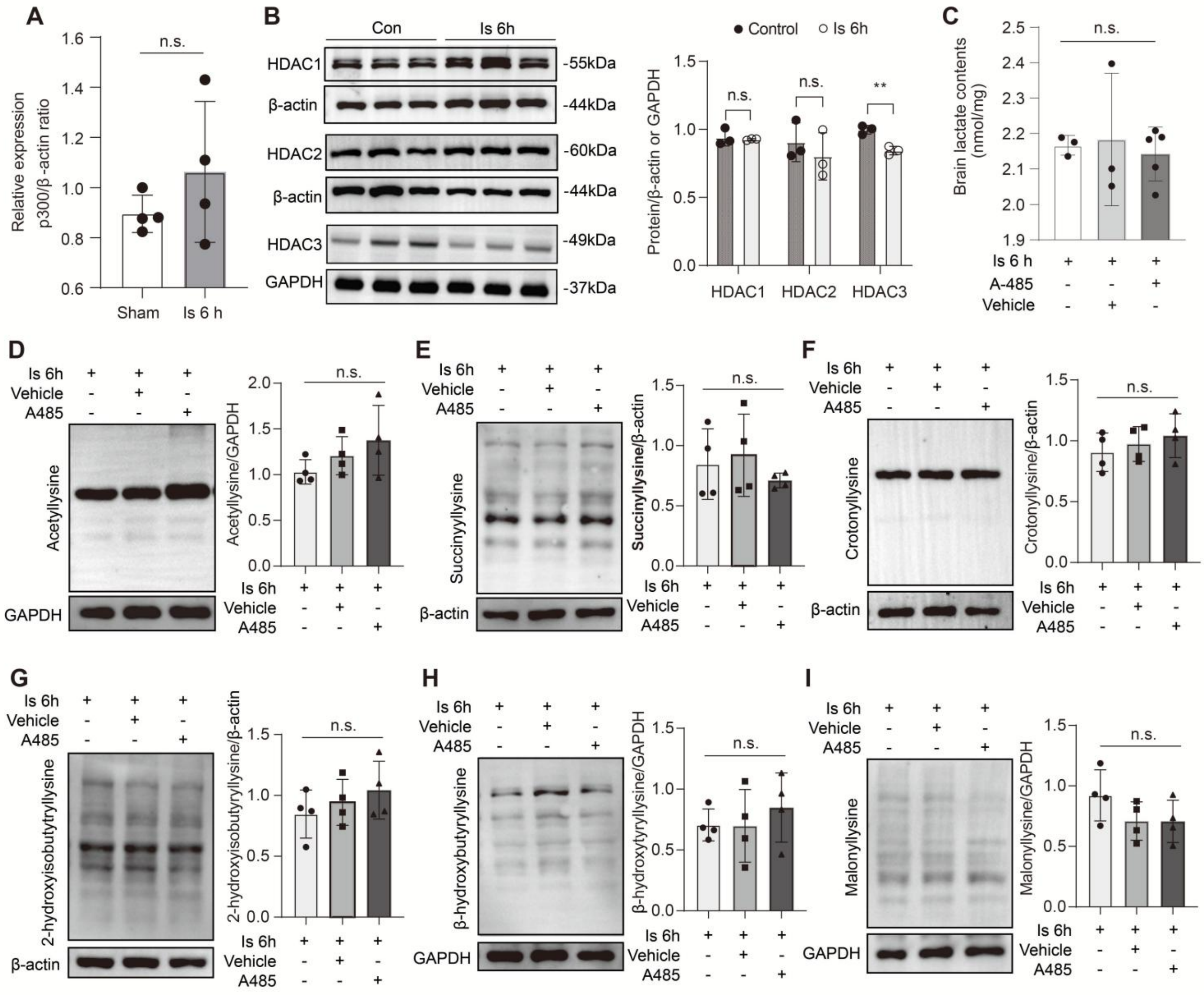

Figure S11. Expression of p300, HDAC1-3 and PTMs after cerebral ischemia. A, The expression of p300 in the brains cerebral ischemia and sham mice detected by qPCR (n = 4). **B,** The representative images and statistical analysis of the expression of HDAC1, 2, and 3 in ischemic brains of mice at 6 h (n = 3). **C,** The ischemic brain levels of lactate at 6 h after cerebral ischemia were measured after A-485 preconditioning (n = 3 - 5). **D-I,** Changes in the expression levels of brain protein acetylation (D), succinylation (E), crotonylation (F), 2-hydroxyisobutyrylation (G), β-hydroxybutyrylation (H), and β-hydroxybutyrylation (I) at 6 h after cerebral ischemia in mice with A-485 preconditioning treatment as detected by the western blot analysis (n = 4). *P < 0.05, n.s. indicates a non-significant difference.

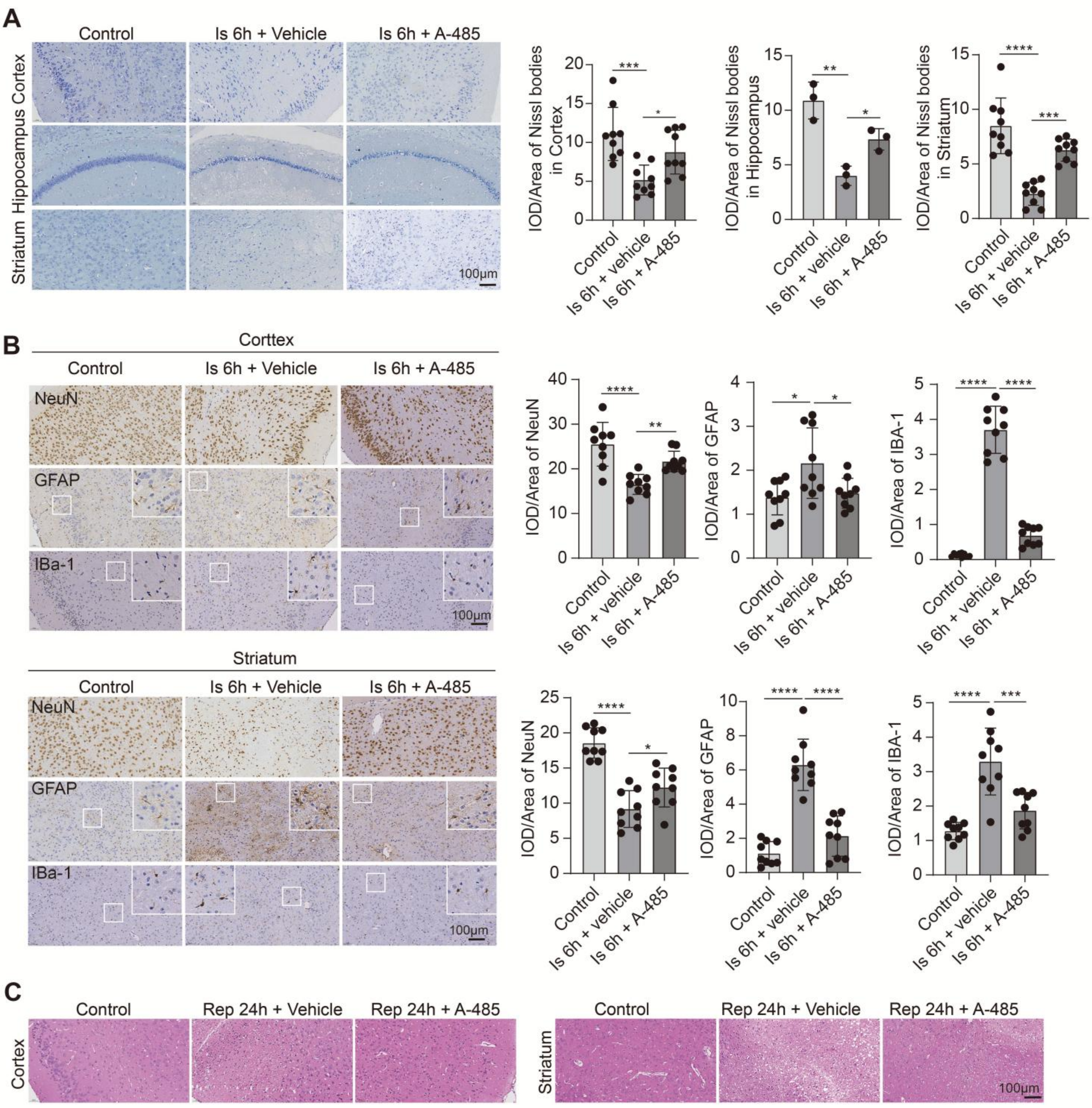

Figure S12. Representative images of brain injury in mice with ischemic stroke preconditioning treated with A-485. A, Representative images and statistical analysis of Nissl staining in the ischemic cortex, hippocampus CA1 region, and striatum at 6 h post cerebral ischemia after A-485 preconditioning (n = 3). **B,** Representative images and statistical analysis of immunohistochemical staining show the expression levels of neuroglial markers of NeuN, GFAP, and IBa-1 in the ischemic cortex and striatum at 6 h post cerebral ischemia after A-485 preconditioning (n = 3). **C,** Representative images of hematoxylin and eosin (H&E) staining show damage in the cortex and striatum at 24

h of reperfusion after 1.5 h ischemia with A-485 preconditioning (n = 3). Scale bar = 100 μm. *$P < 0.05$, **$P < 0.01$, ***$P < 0.001$ and ****$P < 0.0001$.
